# Assessing pedestrian responses to autonomous and personal mobility robots in crowded public spaces

**DOI:** 10.1126/sciadv.aef2576

**Published:** 2026-08-05

**Authors:** Dominik Wojcikiewicz, Aude Billard, Diego Paez-Granados

**Affiliations:** ^1^ Spinal Cord Injury Artificial Intelligence Lab (SCAI), D-HEST, ETH Zürich, Zürich, Switzerland.; ^2^ Digital Health Care and Rehabilitation, Swiss Paraplegic Research (SPF), Nottwil, Switzerland.; ^3^ LASA, Ecole Polytechnique Federale de Lausanne (EPFL), Lausanne, Switzerland.

## Abstract

Robots increasingly share spaces with people, supporting delivery services and mobility for the aging populations, yet their ability to share space comfortably lacks understanding and benchmarks for designers and policy-makers. We compared human-robot (HRI) and human-human (HHI) interactions across four real-world crowd datasets spanning Europe, North America, and Asia, using a unified pipeline to detect interactions, stratify by crowd density, and model pedestrian behavior. Local motion patterns (speed, acceleration, and jerk) remained closely matched between HRI and HHI across all densities. In contrast, proxemics diverged, with effects that grew approximately linearly with robot speed and weakened under higher crowding: In the dataset with the faster navigating robot, pedestrians maintained about 0.23 meters more clearance around the robot than around other pedestrians under less crowded conditions and about 0.05 meters more under more crowded conditions, while in the dataset with the predominantly stationary robot, the corresponding differences were small and inconsistent across crowding levels. The main conclusions were robust to parameter variations and remained stable across a broad range of motion-processing and interaction-labeling settings. Our findings provide density- and speed-aware benchmarks for proxemics in social robot navigation and empirically grounded targets for design, evaluation, and modeling across robotics and urban mobility.

## Teaser

Crowd data show similar motion near humans and robots, but more space around faster robots, especially at lower density.

## INTRODUCTION

Personal space is regulated through proxemics, with Hall describing four distance zones—intimate (0 to 0.45 m), personal (0.45 to 1.2 m), social (1.2 to 3.6 m), and public (greater than 3.6 m)—and emphasizing that preferred spacing depends on situation and culture (*1*). In everyday human-human interaction (HHI) during navigation, these preferences are maintained through anticipatory adjustments: People begin to deflect, reorient, or subtly modulate speed before close approach based on predictions of others’ future motion (*2*–*5*). Prior work modeled pairwise encounters with intended-path updates and reported simple anchors for everyday avoidance: At the closest point of passing, pedestrians typically keep about 0.75 m of distance, and, immediately after passing, they tend to widen the side-by-side gap to at least about 0.8 m (*6*).

Service robots increasingly share these pedestrian-centered spaces, from delivery platforms and cleaning units to personal mobility vehicles (PMVs). Services already available to the public include an autonomous mobility service by WHILL at Narita International Airport, where autonomous PMVs support passenger transfers in departure areas (*7*). As populations age, the number of people with reduced mobility increases, strengthening the case for such assistance in public spaces and transport hubs (*8*). In this context, acceptance and effectiveness depend on how comfortably robots share space with people; measuring when human-robot interaction (HRI) proximities align with or diverge from HHI norms is especially important for PMVs that approach, travel alongside, and, sometimes, dock near people at close range.

Within HRI, initial-encounter studies have examined comfortable distances around a mechanical-looking platform and factors that can influence them. One study reported that many adults stopped in Hall’s personal zone and children in the social zone, with substantial individual variability (*9*). Follow-up work found that a synthesized robotic voice was associated with slightly larger approach distances than natural voices and that prior exposure to the robot reduced standoff distance (*10*). Complementary evidence points to roles for approach direction, speed, and gaze, indicating that context, communicative cues, and familiarity can modulate HRI spacing. Consequently, measured distances may vary across settings, instructions, and prior experience. A recent review and meta-analysis of 27 HRI studies concluded that several frequently discussed moderators, including gender, coarse appearance class, and whether the human or the robot initiated the approach, showed small or inconsistent overall effects, and it called for theory-led, higher-powered studies with better coverage of environmental factors such as crowding and density (*11*).

Motivated by these proxemics and HRI findings, crowd navigation research has advanced beyond purely reactive avoidance toward planners and systems that explicitly anticipate interactions. Learning-based local planners now play a central role in socially compliant collision avoidance and have shown improved robustness across crowd sizes and constrained layouts (*12*–*15*). In parallel, interaction modeling has moved from handcrafted rules toward graph- and prediction-based formulations, including recent heterogeneous interaction graph and transformer architectures for dense scenes (*16*–*18*).

Recent datasets and simulator-based benchmarks have enabled more repeatable comparisons and underscored that performance depends strongly on scenario context, particularly crowd density and environmental geometry (*19*–*22*). Surveys and evaluation-focused reviews highlight persistent gaps in how social navigation is evaluated, noting that common simulation conventions and metrics can miss key aspects of HRI (*23*–*25*). As a result, it remains unclear when and under what conditions pedestrian responses to robots diverge from human-human navigation, especially for service robots such as PMVs in public crowds.

Crowd modeling provides foundations for navigation. Theory indicates that, in the absence of interactions, pedestrians tend to move at nearly constant speed toward their goals (*26*), and the same principle is supported empirically by the strong benchmark performance of the constant velocity model, which captures much of everyday walking with minimal complexity (*27*). Building on this view, deviations in speed and heading and intended-path bending, together with crossing-distance statistics from natural observations (*6*), provide measurable targets for comparing HHI and HRI during navigation.

**Fig. 1.**
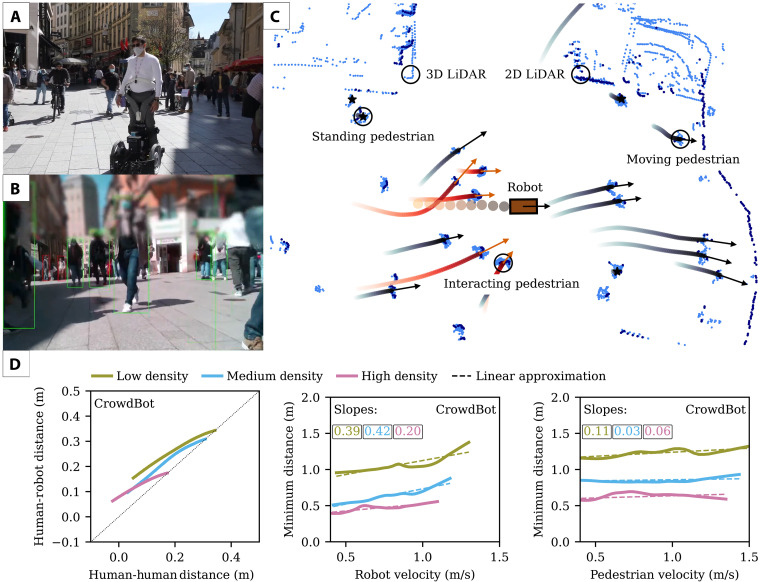
Overview of the evaluation pipeline for the CrowdBot dataset. (**A** and **B**) Scene-level and robot-perspective views, respectively, during dataset recording. (**C**) Bird’s-eye view with tracked pedestrians and the navigating robot during a representative interaction scenario. (**D**) Summary of proxemics differences between HRI and HHI. The leftmost panel shows that pedestrians leave more space to robots than to other humans during close passes, with this effect varying by density. The middle panel shows that average minimum distance to the robot increases approximately linearly with robot speed, with steeper slopes under low- to medium-density conditions. The rightmost panel shows only a weak dependence of spacing on pedestrian speed, which suggests that this effect is robot specific and likely linked to higher-perceived threat at higher speeds.

**Fig. 2.**
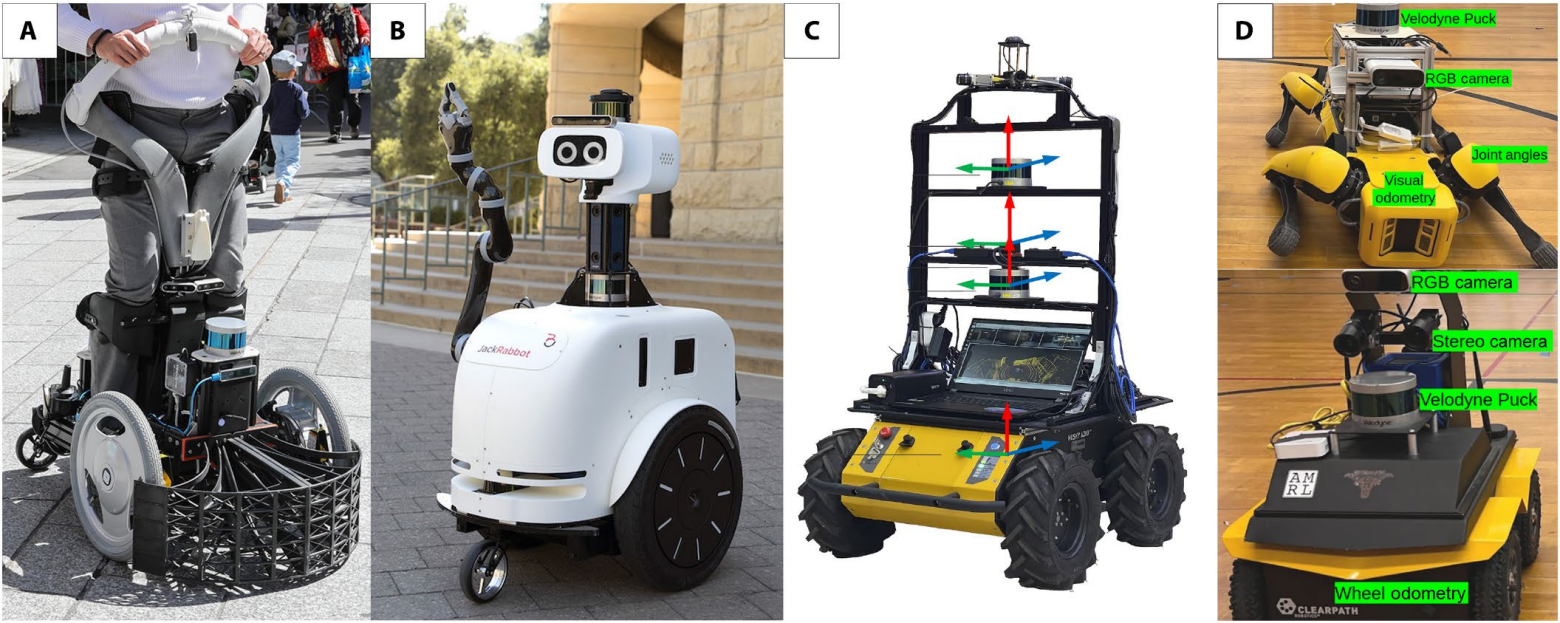
Robots used for data collection in the used datasets. (**A**) Qolo PMV with a person onboard, CrowdBot; (**B**) JackRabbot UGV, JRDB; (**C**) Husky UGV, SiT; (**D**) Spot and Jackal UGVs, SCAND. RGB stands for red, green, and blue.

Here, we examined pedestrian responses to robots in real-world public spaces by comparing HRI and HHI across multiple crowd-navigation datasets. We used four public datasets—CrowdBot (*28*), Socially CompliAnt Navigation Dataset (SCAND) (*20*), JackRabbot Dataset and Benchmark (JRDB) (*29*), and Social Interactive Trajectory (SiT) dataset (*30*)—recorded in Europe, North America, and Asia. The datasets span substantially different pedestrian environments, with CrowdBot recorded in crowded outdoor pedestrian zones in Lausanne, Switzerland; SCAND across a variety of social environments around the University of Texas at Austin campus, USA; JRDB on an indoor-outdoor university campus in Stanford, USA; and SiT in urban scenes in Seoul, South Korea, including streets, hallways, and crosswalks. As a concrete example of these public-space robot-pedestrian scenes, Fig. 1 (A to C) shows a CrowdBot recording from three complementary perspectives: a scene-level view of the robot in a public pedestrian space, a robot-perspective view from the onboard sensing platform, and a bird’s-eye view of tracked pedestrians surrounding the robot. CrowdBot and SCAND are the largest datasets, each providing several hours of data. JRDB is intermediate in size with around 1 hour of data, and SiT is the smallest with only about 15 min of robot navigation. CrowdBot and SCAND provide extensive real-world navigation data but do not include ground-truth pedestrian trajectory annotations, whereas JRDB and SiT are smaller but provide ground-truth pedestrian annotations. The robotic platforms used in these datasets are shown in Fig. 2. While SCAND, JRDB, and SiT use unmanned ground vehicle (UGV) platforms, in CrowdBot, the Qolo robot is a PMV with a user onboard. We treated Qolo as a robot because, in most recordings, the platform is used in shared-control and autonomous navigation modes rather than as a purely passive vehicle. Further technical details, including sensor modalities and dataset characteristics, are summarized in table S1.

The robot-speed regimes also differ substantially across datasets. JRDB is dominated by stationary robot recordings, SiT shows a narrow speed band around 2.8 km hour^−1^, CrowdBot spans a broad range of robot velocities extending up to 4.7 km hour^−1^, and SCAND operates in a higher narrow band around 5.4 km hour^−1^. Pedestrian-speed distributions are broad in all four datasets, but their most common walking speeds vary, increasing from 2.5 km hour^−1^ in JRDB to 3.5 km hour^−1^ in CrowdBot, 4 km hour^−1^ in SiT, and 4.5 km hour^−1^ in SCAND. Based on these dataset characteristics, CrowdBot served as the primary evidence base of this study as it combines natural public-space interactions, broad crowd-density coverage, and the widest robot-speed variation with a large dataset size. A key contribution of this work is CrowdBot-v2 (*31*), a new benchmarking dataset derived from CrowdBot with extensive pedestrian trajectory annotations across multiple hours of robot navigation, which we used throughout the analysis. SCAND and JRDB were used as secondary supporting datasets, with SCAND providing additional large-scale evidence in a higher-speed navigation regime and JRDB providing ground-truth annotations in indoor and outdoor campus scenes. SiT was used as supplementary corroborative evidence in a distinct urban environment, although its smaller number of usable interactions limits the strength of the conclusions that can be drawn from it.

Using light detection and ranging (LiDAR) data from these datasets, we developed a unified pipeline to identify interaction episodes and quantify pedestrian behavior near the robot. This approach allowed us to distinguish local responses associated with nearby encounters from the broader motion patterns of the surrounding crowd. We then quantified behavior along two complementary dimensions: motion dynamics, which capture how pedestrians adjust their movement, and proxemics, which quantify how they regulate spacing relative to others and to the robot. To assess how these responses depend on navigation context, we stratified the analyses by local crowd density and examined their dependence on robot speed. This framework allowed us to test whether robots perturb pedestrian motion, alter interpersonal spacing, or both and whether such effects persist across different crowd conditions, environments, and platforms.

Guided by the literature above, we stated three hypotheses before our analysis:

1) H1 (crowd density conditions). Stratifying environments into low, medium, and high local densities, we expected HRI-HHI differences to be largest at low and medium density and to diminish at higher density as spatial constraints limit pedestrians’ ability to maintain additional separation around the robot (*11*).

2) H2 (robot velocity dependence). We expected pedestrian distance to the robot to increase with robot speed, consistent with higher perceived threat and published effects of speed on comfort (*32*).

3) H3 (motion dynamics difference). As a result of prior works where robots induce different spacing and avoidance behavior than humans, showing larger comfortable distances and speed-sensitive evasive actions around robots (*9*, *32*), we expected that, compared with HHI, HRI elicits stronger motion adaptations, including greater path deflection and transient changes in speed and heading.

Our results provide clear support for H1 and H2, and Fig. 1D previews these patterns at a high level. Consistent with H1, the leftmost panel shows that pedestrians leave more space to robots than to other humans during close passes and that this proxemic difference remains visible over a wider distance range in low- and medium-density conditions than in high-density conditions, where reduced maneuvering freedom compresses spacing. Consistent with H2, the middle and rightmost panels show that pedestrian clearance increases approximately linearly with robot speed, whereas the corresponding dependence on pedestrian speed is weak, suggesting that this effect is primarily robot-specific and consistent with a stronger perceived threat or urgency as robot speed increases. By contrast, we found no evidence supporting H3. Across datasets and analyses, motion dynamics differ only weakly between HRI and HHI, indicating that the clearest divergence between pedestrian responses to robots and to humans lies not in large local kinematic disturbances, but in how space is allocated around the robot. This combination of pronounced proxemic differences and only small motion-dynamics differences is consistent with the anticipatory nature of human navigation in public spaces. In this view, small early motion adjustments can accumulate into larger spacing differences during encounters, reinforcing recent work that emphasizes anticipation as a key mechanism underlying coordinated collision avoidance in human crowds. Together, these findings provide previously unavailable empirical evidence that robot-specific responses in public-space navigation are expressed most clearly through density-aware and speed-dependent proxemics, and they establish practical benchmarks for studying, modeling, and evaluating socially compatible robot behavior in crowded public environments.

## RESULTS

### Analysis setup and key definitions

**Fig. 3.**
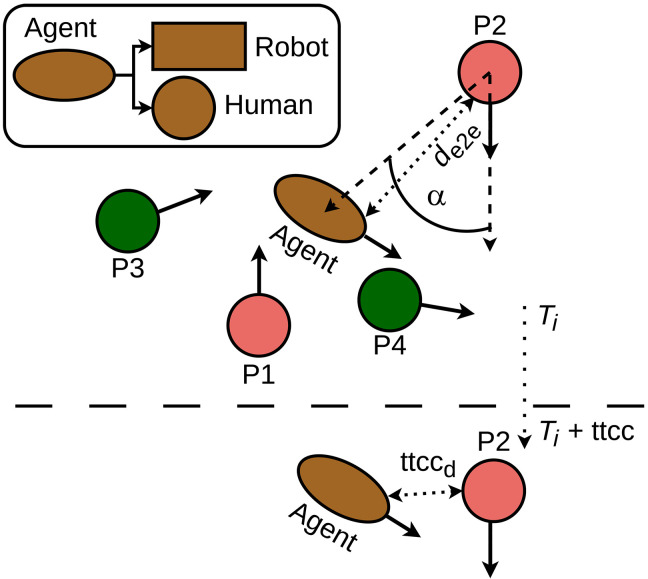
Visualization of interaction labeling. In the example frame, pedestrians P1 and P2 (red) are labeled as interacting and P3 and P4 (green) as noninteracting relative to the agent. In HRI, the agent is a robot, modeled as a rectangle, whereas, in HHI, it is a human, modeled as a circle. Labels require both conditions: $\alpha \leq \alpha_{\text{dyn}}\left(d_{e2e}\right)$ for perception and ttcc≤3 s for imminent proximity, where α is the heading-to-agent angle, $d_{e2e}$ is edge-to-edge separation, and ttcc is the constant-velocity time to reach ${\text{ttcc}}_{d}$ of 0.8 m.

For the analyses reported below, we first partitioned pedestrians with respect to the robot into two complementary subsets: HRI-ped and NI-ped. HRI-ped denotes pedestrians during frames labeled as interacting with the robot, whereas NI-ped denotes pedestrians during frames labeled as noninteracting with the robot and thus serves as a crowd baseline without an assigned robot interaction. Interaction labels were assigned agent-centrically: A pedestrian was labeled as interacting when, relative to a designated agent, the pedestrian both perceived that agent and was on a short-horizon near-collision course. Separately, to quantify how pedestrians interact with specific other pedestrians rather than only with the crowd as a whole, we constructed the HHI-ped subset by selecting human agents within each recording and applying the same interaction-labeling rule with respect to them. Similarly to HRI-ped, HHI-ped denotes pedestrians during frames labeled as interacting with those selected human agents. Figure 3 visualizes this shared interaction-labeling rule for a navigating agent and shows how interacting frames are defined in both the HRI and HHI settings.

To assess how pedestrian responses depend on local crowding, we stratified the analyses into three density regimes—low, medium, and high—using local crowd-density features and *k*-means clustering. The resulting centroid densities, expressed in people per square meter (ppsm), were (0.19, 0.10) for low density, (0.49, 0.28) for medium density, and (0.78, 0.50) for high density, where each pair denotes the maximum and average local density experienced during a trajectory. Across the analyzed datasets, the observed densities remained below about 0.90 ppsm, placing the reported interactions within the anticipatory and cooperative crowd regimes.

We quantified pedestrian responses along two complementary dimensions. Motion dynamics were characterized using angular velocity, linear acceleration, and linear jerk derived with finite differences. For angular velocity and linear acceleration, we used a 1-s window to capture changes in orientation and speed at the scale of two human steps. For linear jerk, which is intended to capture more sudden interactions and events, we used a shorter 0.5-s window. To examine individualized effects, we also compared interacting and noninteracting segments for pedestrians observed for at least 1.5 s in both contexts and normalized these differences by each pedestrian’s trajectory-wide mean. Last, to examine more reactive behavior, we analyzed significant events, defined as interactions in which either the turn angle exceeds 45° or the velocity change exceeds 2 km hour^−1^ within 1 s. Proxemics were characterized using minimum edge-to-edge distance near closest approach and by counting intrusions into a 0.3-m comfort zone around the reference agent. For the minimum-distance analysis, we compared the robot-centered nearest-pedestrian distance distribution with the corresponding pedestrian-centered baselines HRI-ped and NI-ped within each density stratum. We summarized the shifts between these distributions using the close-pass regime and the maximum excess clearance Δ within that regime.

To reduce artifacts from short or fragmented trajectories, trajectory-based analyses were restricted to tracks lasting at least 3.5 s. After further restricting the motion analyses to moving pedestrians in the robot near field, the retained samples comprised about 12,000 pedestrians in CrowdBot, 14,000 in SCAND, 900 in JRDB, and 100 in SiT. Stationary pedestrians and shorter tracks were retained only for proxemics calculations. For the proxemics analyses, we further applied a comotion exclusion intended to remove within-group proxemics. Concretely, this exclusion removes persistently comoving pairs whose distance changes by less than 2 m over their shared frames while accepting that it also removes some nongroup cases such as sustained following. To minimize differential bias across conditions, the same exclusion was applied to both pedestrian-pedestrian and pedestrian-robot pairs.

Further implementation details are provided in Materials and Methods, including the full interaction-labeling algorithm, the selection of human agents for the HHI-ped analysis, the density-stratification procedure, the motion-processing framework, the proxemics definitions including the close-pass regime and maximum excess clearance Δ, and the filtering pipeline with pedestrian eligibility criteria and comotion exclusion. Supporting sensitivity analyses are provided in the Supplementary Materials.

### Motion dynamics similarity between HRI and HHI

**Fig. 4.**
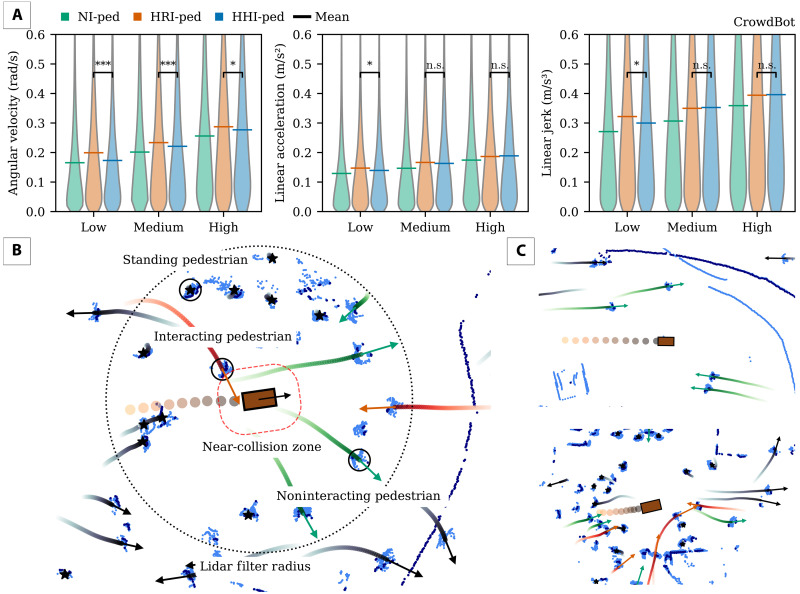
Motion dynamics for the NI-ped, HRI-ped, and HHI-ped groups in CrowdBot. (**A**) Distributions of angular velocity, linear acceleration, and linear jerk per pedestrian, grouped by local density. Horizontal lines denote group means for visual orientation. Asterisks indicate the statistical significance of HRI-ped versus HHI-ped comparisons within each density stratum (***P<0.001; *P<0.05; n.s., P>0.05). Effect sizes were assessed as distribution shifts using Wasserstein distances and are reported in the main text. (**B**) Overhead view of a representative medium-density scenario with interaction annotations. (**C**) Example frames illustrating low-density (top) and high-density (bottom) conditions.

We assessed whether robot encounters perturb local pedestrian kinematics beyond effects caused by other pedestrians in the crowd by comparing angular velocity, linear acceleration, and linear jerk across HRI-ped, HHI-ped, and NI-ped groups stratified by crowd density. In CrowdBot, the differences in pedestrian motion dynamics (Fig. 4A) between HRI-ped and HHI-ped remained small across all density regimes. For angular velocity, the Wasserstein distances between HRI-ped and HHI-ped were below 0.03 rad s^−1^ in all density conditions, corresponding to less than about 2° difference per second. For linear acceleration, the Wasserstein distances remained below 0.01 m s^−2^, corresponding to a speed-change difference of less than about 0.04 km hour^−1^ per second. For linear jerk, they remained below 0.03 m s^−3^, again indicating only negligible distribution shifts. Thus, although some pairwise comparisons reached statistical significance because of the large CrowdBot sample size, the distribution-level differences between HRI-ped and HHI-ped remained small throughout. As density increased, the HRI-ped and HHI-ped distributions remained closely matched, consistent with the view that reduced maneuvering freedom and behavioral alignment in denser crowds limit any remaining response differences.

Similar patterns were observed in the SCAND, JRDB, and SiT datasets (Supplementary Text). SCAND, which represents a larger sample size and a higher-speed robot regime, also showed closely matched HRI-ped and HHI-ped motion-dynamics distributions. JRDB and SiT, although smaller, provide ground-truth pedestrian annotations and did not suggest a clear or conclusive HRI-ped versus HHI-ped difference.

### Individualized motion changes and significant events

**Fig. 5.**
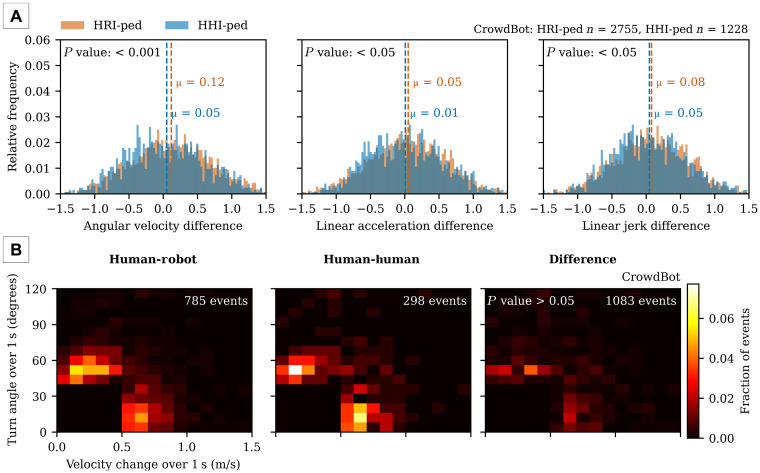
Individualized motion changes and significant events in CrowdBot. (**A**) Normalized per-pedestrian motion changes relative to trajectory-wide behavior in the HRI-ped and HHI-ped conditions. Top left of each panel reports the *P* value for the HRI-ped versus HHI-ped comparison, and the annotated μ values indicate the corresponding means. Across angular velocity, linear acceleration, and linear jerk, the normalized changes remained small, indicating limited influence of interaction type on individual-level motion adaptation. (**B**) Distributions of significant events for HRI and HHI in the joint space of velocity change and turn angle measured over 1 s during an interaction event. The rightmost panel shows the residual between the two event maps. Differences remained small across the event space, with no significant multivariate distributional difference (P>0.05).

To determine whether the small group-level differences in motion metrics reflect consistent behavioral shifts at the level of individual pedestrians, we asked whether pedestrians change their motion in a similar way when interacting with a robot or with another pedestrian, relative to their own trajectory-wide behavior. In CrowdBot, across all metrics, the distributions of these normalized interaction-related motion changes remained closely matched between HRI-ped and HHI-ped with Cohen’s *d* = 0.11, 0.07, and 0.06 for angular velocity, linear acceleration, and linear jerk, respectively, indicating that interaction type had only a limited influence on individual-level motion (Fig. 5A).

We next examined whether this similarity also holds for sudden, more reactive events with larger magnitudes of velocity or orientation changes. In CrowdBot, event density maps for HRI-ped and HHI-ped were similar across the event space (Fig. 5B), and a two-sample multivariate energy-distance test on the paired event features did not detect a difference between HRI-ped and HHI-ped events. These results reinforce the consistency of pedestrian responses across interaction types, even during sudden and urgent adjustments.

Analogously to the group-level motion results, the individualized-change and significant-event analyses on SCAND, JRDB, and SiT led to the same qualitative conclusion and did not indicate a clear HRI-ped versus HHI-ped difference (Supplementary Text).

### Close-pass clearance is greater around moving robots

**Fig. 6.**
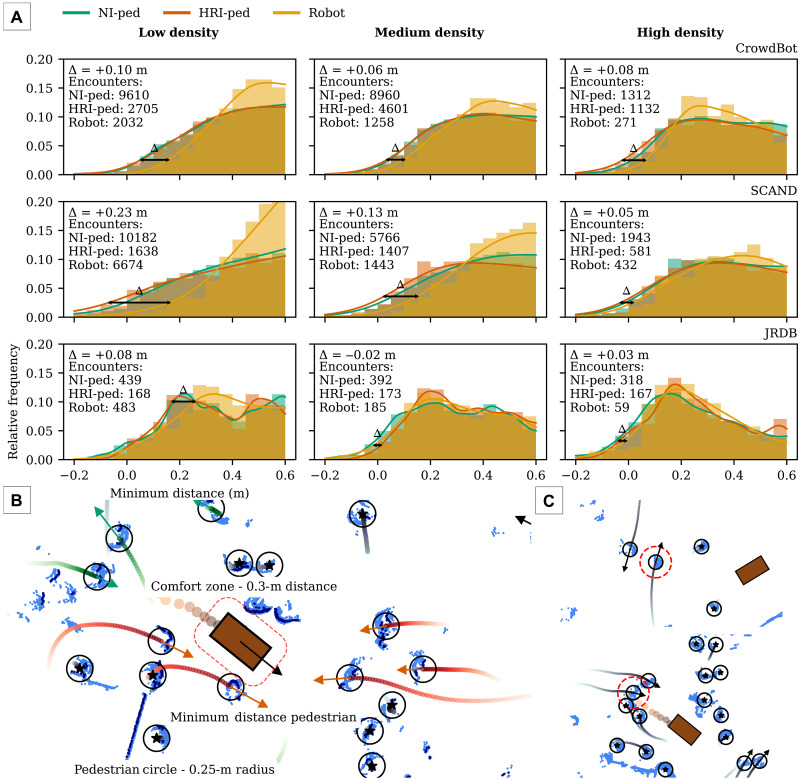
Minimum-distance analysis. (**A**) Kernel density estimates of minimum edge-to-edge distances. Larger clearances around Robot relative to HRI-ped and NI-ped are most pronounced in SCAND, followed by CrowdBot, and are weak and inconsistent in JRDB, matching the different robot-speed regimes represented by these datasets. Across density strata, the robot-centered excess generally contracts under higher crowding, suggesting that surrounding pedestrian constraints reduce both the additional clearance maintained around the robot and the distance range over which this separation remains visible. (**B**) Overview showing robot comfort-zone boundary and pedestrian perimeters. (**C**) Low-density (top) and high-density (bottom) pedestrian crossing scenarios.

We next examined whether pedestrians leave different clearances around robots than around humans by comparing minimum-distance distributions across density strata and datasets (Fig. 6A). Across datasets, the HRI-ped and NI-ped distributions were largely aligned, indicating that any change in spacing around the robot does not come at the cost of diminished spacing to other pedestrians. The main proxemic difference instead appeared in the robot-centered distributions. In CrowdBot and SCAND, the Robot curve was consistently shifted toward larger minimum distances than the corresponding pedestrian-centered baselines, whereas, in JRDB, this effect was much weaker and less consistent.

This cross-dataset pattern is consistent with the different robot-motion regimes represented in the three datasets. In JRDB, where the robot is predominantly stationary, the clearance around the robot approached that of other pedestrians and did not remain consistently separated. In CrowdBot, where the robot spans a broad range of operating speeds, the robot-centered clearance shift was more pronounced, with Δ = +0.10, +0.06, and +0.08 m in low-, medium-, and high-density conditions, respectively. In SCAND, where the robot mostly operates around 5.4 km hour^−1^, the effect was strongest, reaching Δ = +0.23 m in low density and remaining positive at +0.13 and +0.05 m in medium and high densities.

Robot appearance may also contribute to these cross-dataset differences. JackRabbot in JRDB (Fig. 2B) has a rounded, more approachable appearance, whereas Qolo in CrowdBot (Fig. 2A) resembles a PMV with a user onboard, and the SCAND robots (Fig. 2D) have sharper edges and more exposed hardware. Although these appearance differences cannot be isolated in the present data, they are consistent with the possibility that pedestrians were more willing to pass close to JackRabbot, somewhat less so to Qolo, and least so to the SCAND platforms. We therefore interpret the cross-dataset differences primarily in relation to robot-speed regime while noting robot appearance as a plausible additional factor.

Across density strata, the clearest pattern is visible in SCAND. There, Δ shrank from 0.23 m in low density to 0.05 m in high density, consistent with the interpretation that pedestrians have less freedom to realize their preferred robot clearance as crowding increases. CrowdBot showed the same general tendency, but more clearly through the extent rather than the magnitude of the close-pass separation. In low- and medium-density conditions, the robot-centered excess persisted until ∼0.35 m, whereas, in high density, it disappeared already by ∼0.20 m, a pattern also previewed in Fig. 1D. SCAND showed the same contraction of the close-pass regime, although somewhat less distinctly. Together, these results suggest that increasing density acts in two related ways: It reduces the amount of additional clearance pedestrians maintain around a moving robot, and it shortens the distance range over which this robot-specific clearance remains visible. SiT yielded too few usable encounters for a robust main-text comparison and is, therefore, reported in the Supplementary Text.

### Intrusions into the comfort zone are less frequent around robots

**Fig. 7.**
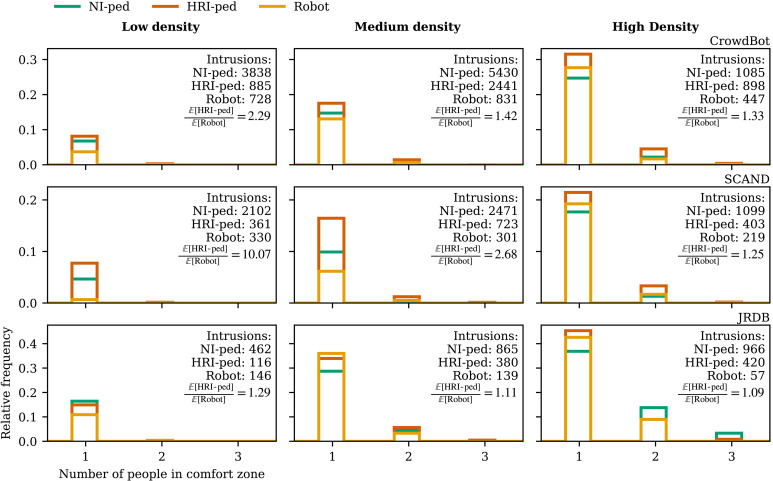
Intrusions into agent comfort zones across densities. Relative frequencies of observing pedestrians inside the 0.3-m comfort zone around each agent. Panel annotations report total intrusion counts and the ratio of mean intrusion counts E[HRI−ped]/E[Robot]. Across datasets, robots experience fewer comfort-zone intrusions than nearby pedestrians, with the effect strongest in SCAND, intermediate in CrowdBot, and weakest in JRDB. This difference decreases with density, indicating that higher crowding reduces pedestrians’ ability to preserve additional clearance around robots.

Building on the minimum-distance analysis, we next quantified close passes by counting how often other pedestrians entered the 0.3-m comfort zone around each agent (Fig. 7), a threshold motivated by prior natural-observation work on close pedestrian encounters (*6*). This discrete intrusion analysis complements the continuous minimum-distance distributions by reducing all encounters below the 0.3-m comfort-zone threshold to a single summary metric. It therefore captures the overall strength of the robot-specific spacing effect below that cutoff, without depending on where within that range the largest distributional difference occurs.

Across all datasets and density strata, robots experienced fewer comfort-zone intrusions than nearby pedestrians in the HRI-ped subset, despite the robot’s larger physical footprint. This pattern mirrors the minimum-distance analysis and again indicates that pedestrians preserve additional space around robots when conditions allow. The strength of the effect was strongly dataset dependent. SCAND showed the largest difference, with E[HRI−ped]/E[Robot]=10.07,2.68, and 1.25 in low-, medium-, and high-density conditions, respectively. CrowdBot showed an intermediate effect, with corresponding ratios of 2.29, 1.42, and 1.33. JRDB showed the weakest effect, with ratios remaining below 1.30 across all densities. Thus, as in the minimum-distance analysis, the largest robot-specific proxemic differences appear in SCAND, followed by CrowdBot, and are weakest in JRDB.

Across all three datasets, the intrusion-count ratio decreased with increasing density. This provides further evidence that higher crowding progressively reduces the additional clearance maintained around robots and forces pedestrians to move closer to them. As in the minimum-distance analysis, SiT is reported in the Supplementary Text.

### Spacing increases linearly with robot speed

**Fig. 8.**
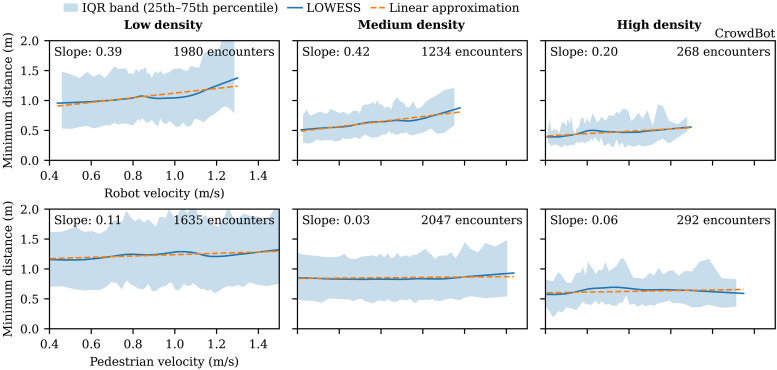
Minimum distance versus robot and pedestrian speed across crowd densities. The top row shows minimum distance as a function of robot speed, and the bottom row shows the corresponding pedestrian-speed control. Blue curves denote locally weighted scatterplot smoothing (LOWESS) estimates, orange dashed lines are linear approximations to the LOWESS trends, and blue bands indicate the interquartile range (IQR). Minimum distance increases approximately linearly with robot speed, with steeper slopes at low and medium density, whereas the corresponding dependence on pedestrian speed is much weaker.

We next assessed in detail how speed shapes pedestrian spacing by relating minimum edge-to-edge distance to instantaneous robot velocity within each density stratum and comparing this effect with an analogous pedestrian-speed control (Fig. 8). In CrowdBot, minimum distance increased approximately linearly with robot speed, with slopes of 0.39, 0.42, and 0.20 m (m s^−1^)^−1^ in low-, medium-, and high-density conditions, respectively. The weaker slope in high density is consistent with stronger surrounding constraints that limit pedestrians’ ability to adjust their distance from the robot.

By contrast, the corresponding dependence on pedestrian speed was much weaker, with slopes of only 0.11, 0.03, and 0.06 m (m s^−1^)^−1^ across the same density strata. Thus, while minimum distance increases systematically with robot speed, it changes only weakly with pedestrian speed. This pattern suggests that the speed dependence observed in the top row is not merely a generic effect of faster motion but is primarily robot specific and consistent with a stronger perceived threat or urgency as robot speed increases.

This interpretation is also supported by the cross-dataset proxemics analyses above. The weakest robot-specific spacing effects were observed in JRDB, where the robot is predominantly stationary; the intermediate effects were observed in CrowdBot, where robot speeds span a broad range; and the strongest effects were observed in SCAND, where the robot typically operates at higher speed. Together, the within-dataset linear trend in CrowdBot and the ordering of proxemic differences across datasets both support the conclusion that robot speed is an important determinant of pedestrian clearance in public-space navigation. We restricted this speed-dependence analysis to CrowdBot because it is the only dataset in this study that combines a broad range of robot velocities with a sufficient number of close-pass encounters across density strata.

## DISCUSSION

### Summary of findings

Proxemics, rather than local motion dynamics, most clearly differentiated HRI from HHI. Across datasets, pedestrians maintained greater close-pass clearance around robots than around humans, with the effect strongest in SCAND, intermediate in CrowdBot, and weakest and least consistent in JRDB. In CrowdBot, the robot-centered excess remained visible up to ∼0.35 m in low and medium density and up to ∼0.20 m in high density. In SCAND, the maximum excess clearance Δ decreased from 0.23 m in low density to 0.13 m in medium density and 0.05 m in high density. Intrusions into the comfort zone followed the same pattern across CrowdBot, SCAND, and JRDB, with fewer intrusions around robots than around nearby pedestrians and a progressive reduction of this difference as density increased. Within CrowdBot, minimum distance increased approximately linearly with robot speed, with slopes of 0.39, 0.42, and 0.20 m (m s^−1^)^−1^ in low-, medium-, and high-density conditions, respectively, whereas the corresponding dependence on pedestrian speed was much weaker. By contrast, we did not observe a substantial difference in motion dynamics between HRI and HHI. Angular velocity, linear acceleration, and jerk remained closely matched across conditions at the group level, in individualized normalized changes, and in significant-event analyses.

### Anticipatory reallocation of space

The combination of distinct spacing and similar kinematics points to an anticipatory pedestrian response to robots in public spaces. Pedestrians appear to resolve potential conflicts early through modest adjustments in path and timing that preserve smooth trajectories while increasing clearance around the robot. This finding is contrary to H3, which predicted stronger local motion adaptations in HRI than in HHI. Instead, the motion metrics remained similar across interaction types, suggesting that the clearest difference between HRI and HHI lies not in how abruptly pedestrians move, but in how much clearance they preserve around the robot during encounters. This interpretation is consistent with recent work emphasizing anticipation as a key mechanism underlying coordinated collision avoidance in human crowds.

### Density-dependent proxemics

Availability of free space governed the expression of robot-human spacing. When density was low or moderate, pedestrians maintained more additional clearance around the robot, and this separation extended over a broader close-pass regime. Under stronger crowding, this excess contracted. Our results suggest that density acts in two related ways: It reduces how much additional clearance pedestrians maintain around the robot, and it shortens the distance range over which this robot-specific separation remains visible. This pattern supports H1, which predicted that robot-human spacing differences would be more pronounced at lower densities and diminish under crowding due to spatial constraints. It is also consistent with prior findings that human-robot spacing tends to exceed human-human spacing, with the magnitude depending on context (*9*, *11*).

### Speed-dependent spacing

Robot speed shaped proxemics within each density stratum. In CrowdBot, higher robot speeds were associated with larger clearances, whereas the corresponding dependence on pedestrian speed was much weaker. This control comparison makes a generic faster-agent explanation unlikely and instead points to a robot-specific speed effect. The same interpretation is supported by the cross-dataset ordering of proxemic differences: The weakest effects were observed in JRDB, where the robot is predominantly stationary; the intermediate effects in CrowdBot, where robot speeds span a broad range; and the strongest effects in SCAND, where the robot typically operates at higher speed. These findings support H2 and are consistent with the interpretation that faster robot motion is associated with greater perceived urgency or threat.

### Dataset and context differences

Recording context, platform, and motion profiles differed substantially across datasets. JRDB involved a mostly stationary UGV in a university-campus setting, CrowdBot involved a PMV with a rider navigating dense public pedestrian zones, and SCAND involved faster-moving mobile platforms in a different social-navigation regime. These differences help explain why the proxemic effects are not identical across datasets, but they also introduce covariation that cannot be disentangled here. In particular, robot-speed regime appears to be the clearest common factor, but platform appearance, rider presence, and environmental context may also contribute. For example, JackRabbot has a more rounded and approachable appearance, whereas Qolo and, especially, the SCAND robots present a more vehicle-like or industrial form. These covariations motivate controlled cross-site studies that manipulate platform, speed, and setting independently.

### Limitations

Because this study used real-world recordings rather than controlled experiments, differences associated with robot speed, appearance, rider presence, and environment cannot be interpreted as independent causal effects. Pedestrians were approximated as circles and robots as rectangles, which introduces geometric approximation error in edge-to-edge distance, particularly at small separations. This limitation mainly affects the closest-pass proxemics analyses, where it can produce slight negative distances or modest shifts in minimum-distance and intrusion estimates, but it is less relevant to the motion-dynamics comparisons and the broader density- and speed-dependent trends. Quantifying the exact magnitude of this uncertainty requires a higher-fidelity body-shape representation and is left for future work. The proxemics analyses also used a comotion exclusion intended to remove likely within-group encounters without inferring true social-group membership from trajectories. While this exclusion reduced bias from within-group proxemics by removing most pairs likely to reflect within-group comotion, it also removed some nongroup interactions, such as sustained following. Sample sizes varied by dataset and density stratum, robot motion in JRDB was limited, and SiT provided too few close-pass encounters for robust proxemics inference in the main text. Generalization beyond the studied settings, platforms, and cultural contexts, therefore, remains to be established.

### Implications and outlook

The results provide density-aware and speed-aware benchmarks for evaluating navigation in mixed human-robot traffic. For evaluation, they suggest that proxemic criteria are more sensitive than local motion-dynamics metrics for distinguishing HRI from HHI under natural conditions. For robot design and control, they suggest that moderating operating speed in uncongested areas may reduce the additional clearance pedestrians feel compelled to maintain around the robot. For modeling, the findings support anticipatory planners that couple smooth, human-like motion with proximity-sensitive objectives, rather than relying only on reactive avoidance cues or local kinematic disturbance measures. Future work should expand platform diversity, include explicit social signaling, and perform controlled cross-site experiments that decouple environment, platform, rider presence, and speed while holding sensing and analysis constant.

## MATERIALS AND METHODS

### Data preprocessing, detection, and tracking

Pedestrians were detected using Person-MinkUNet (*33*) for three-dimensional (3D) point clouds and DR-SPAAM (*34*) for 2D LiDAR scans. Both models were trained on the JRDB dataset. Valid detections were selected using thresholds of 0.5 (3D) and 0.75 (2D) to reduce false positives.

To track individuals over time, the detections were passed on to the AB3DMOT tracker (*35*), which uses a Kalman filter with a Hungarian algorithm for data association. Detection coordinates were transformed into a global reference frame to isolate pedestrian movement from robot movement.

For data association, we used a sequential matching approach based on 3D/2D intersection over union (IoU) followed by 3D/2D distance metrics. Initially, associations were made using IoU, with thresholds set at 0.3 for 2D and 0.33 for 3D IoU. The remaining unmatched detections were then assigned on the basis of their spatial distance, with a threshold of 0.5 m for both 3D and 2D detections.

To integrate both 2D and 3D detections for tracking, we used a range-based merging strategy. Within 5 m of the robot, 2D detections were used and 3D detections within this range were projected into 2D. The tracking was then performed using a 2D IoU/distance-based association method. Beyond 5 m, only 3D detections were used, with the tracking based on the 3D IoU/distance-based association.

### Motion data derivation

For the CrowdBot and SCAND datasets, pedestrian trajectories were obtained from the tracking pipeline described above, whereas for JRDB and SiT, ground-truth annotations were used. After extracting pedestrian position trajectories, we retained only tracks lasting at least 3.5 s for motion analysis.

Motion analyses considered only moving pedestrians. A pedestrian was classified as moving if their speed exceeded 0.6 m s^−1^ for at least 1 s during the track. For pedestrians classified as moving, only frames with speed above 0.4 m s^−1^ were retained for motion-metric derivation, thereby excluding bystanders. Stationary pedestrians and shorter tracks were retained only as passive neighbors for proxemics calculations.

To reduce measurement noise while preserving trajectory structure, the position data were smoothed using a Savitzky-Golay filter with a cubic polynomial and a 1.5-s window.

Kinematic quantities were then derived from finite differences chosen to match interpretable gait time scales. Linear velocity, linear acceleration, and angular velocity were computed over a 1-s window, which corresponds approximately to two human steps and captures changes in speed and heading at the scale of ordinary walking adaptation. Linear jerk, intended to capture more abrupt short-horizon adjustments, was computed over a shorter 0.5-s window. For angular velocity, the instantaneous movement direction was estimated from the velocity trajectory.

To support the validity of these motion metrics, we performed two sensitivity analyses. First, we compared predicted and annotated trajectories on JRDB to confirm that model-based tracking does not introduce systematic bias into the motion metrics. Second, we verified that the main motion-dynamics results were robust to the choice of motion-processing parameters. Both analyses are reported in the Supplementary Text.

### Framewise interaction labeling

Pedestrian trajectories were partitioned framewise into interacting and noninteracting subsets using the same interaction-labeling rule in both the HRI and HHI settings. In HRI, the designated agent was the robot, and, in HHI, it was a selected human agent. Labels were assigned agent-centrically: A pedestrian frame was labeled as interacting when, relative to the designated agent, the pedestrian both perceived that agent and was on a short-horizon near-collision course. Interaction was, therefore, defined relative to the designated agent and did not require reciprocal labeling.

#### Dynamic angle threshold

Perception was evaluated using the angle α between the pedestrian heading and the vector pointing to the designated-agent center. This angle was compared with a distance-dependent dynamic threshold $\alpha_{\text{dyn}}\left(d_{e2e}\right)$, where $d_{e2e}$ is the edge-to-edge distance between the pedestrian and the designated agent. The threshold was linearly interpolated between 35° and 100°. For $d_{e2e}<0.5\;m$, the threshold was fixed at 100°, reflecting broader spatial awareness at close range. For $d_{e2e}>3.0\;m$, it was fixed at 35°, reflecting a narrower forward-view criterion at larger separation. In HRI, the designated agent was modeled as an oriented rectangle, and, in HHI, the designated agent was modeled as a circle.

#### Time to close collision

Imminent proximity was evaluated using the time to close collision (ttcc), defined as the time remaining until the pedestrian and the designated agent would reach an edge-to-edge distance of 0.8 m under constant-velocity motion.

#### Interaction assignment and extension

A frame was labeled as interacting only if both cues were satisfied simultaneously

$$\alpha \leq \alpha_{\text{dyn}}\left(d_{e2e}\right)\;\text{and}\;\text{ttcc}\leq 3\;s$$ (1)

To capture the full human response after passing, the end of each detected interaction was extended by 2 s.

#### Framewise partitioning and near-field filter

Because the partitioning was performed framewise, different segments of the same pedestrian trajectory could belong to the interacting and noninteracting subsets. Noninteracting frames were further restricted to pedestrians whose distance to the robot center was less than 5 m, to reduce the influence of occlusions and reduced detection confidence at larger range.

#### Sensitivity analysis

To assess the robustness of the interaction-labeling rule, we performed a sensitivity analysis across seven parameter configurations spanning more reactive to more anticipatory definitions. The results are reported in the Supplementary Text and show that the main patterns remain stable across these choices.

The partitioning process is detailed in Algorithm 1 and illustrated in Fig. 3.

**Algorithm 1.**
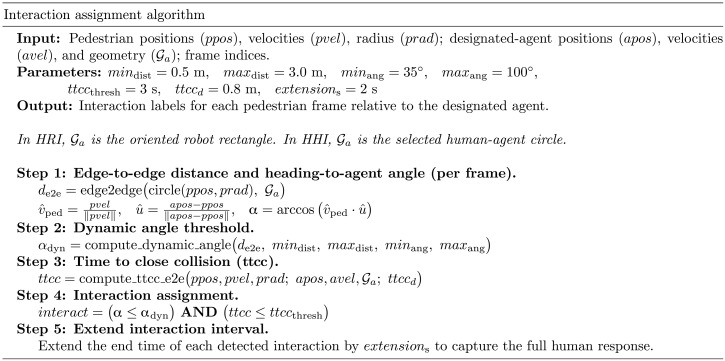
Framewise interaction assignment relative to a designated agent. Frames are marked as interacting when two cues are satisfied simultaneously: perception and imminent proximity. Perception is tested by comparing the heading-to-agent angle α to a distance-dependent threshold $\alpha_{\text{dyn}}\left(d_{e2e}\right)$, where $d_{e2e}$ is the edge-to-edge separation between the pedestrian and the designated agent. Imminent proximity is tested via the time to close collision (ttcc), defined as the constant-velocity time for $d_{e2e}$ to reach 0.8 m. A frame is labeled as interacting only if $\alpha \leq \alpha_{\text{dyn}}$ and $\text{ttcc}\leq {\text{ttcc}}_{\text{thresh}}$. The interaction is then extended by ${\text{extension}}_{s}$ to capture the full human response. In HRI, the designated agent was the robot, and, in HHI, it was a selected human agent.

### Human-human control selection

To construct the human-human control, selected pedestrians within each sequence were treated as designated human agents and processed analogously to the robot in the HRI analysis. The selection procedure was as follows: (i) identify eligible pedestrians with at least 3.5 s of frames in the noninteracting subset; (ii) rank the eligible pedestrians by the number of noninteracting frames in which their speed exceeded 0.4 m s^−1^, corresponding to their moving frames; and (iii) select the top-ranked pedestrians until either the total number of selected moving frames exceeds the number of frames during which the robot navigated in the same sequence, or the list of eligible pedestrians is exhausted. The selected pedestrians were then used as designated human agents for constructing the HHI-ped subset with the same interaction-labeling rule used in HRI.

### Crowd density stratification procedure

To investigate how behavioral metrics vary with local crowding, we stratified trajectories into three density regimes: low, medium, and high. The stratification was derived on CrowdBot using weighted *k*-means++ clustering with *k* = 3.

For each pedestrian trajectory, we computed two local-density features within a 2.0-m social zone: the maximum crowd density and the average crowd density encountered during the trajectory. These two features define a point in a 2D density space for each pedestrian.

To prevent frequently sampled density ranges from dominating the clustering, trajectories were first binned by maximum crowd density, and each bin was assigned the same total weight. This ensured that sparsely sampled density ranges contributed comparably to the clustering and reduced overrepresentation of common low- and medium-density cases. To further mitigate the influence of outliers and unreliable cases, we excluded any point corresponding to the line (0, 0) as well as those lines derived from fewer than 20 observed individuals. This helped prevent sparse and anomalous data from distorting the boundary of the density group.

For encounter-level analyses, density labels were also assigned to the robot. The robot trajectory was divided into 4-s segments, and the local pedestrian density around the robot was computed within each segment using the same two features. These robot segments were then classified into the previously defined pedestrian-based density strata using the same clustering boundaries.

**Fig. 9.**
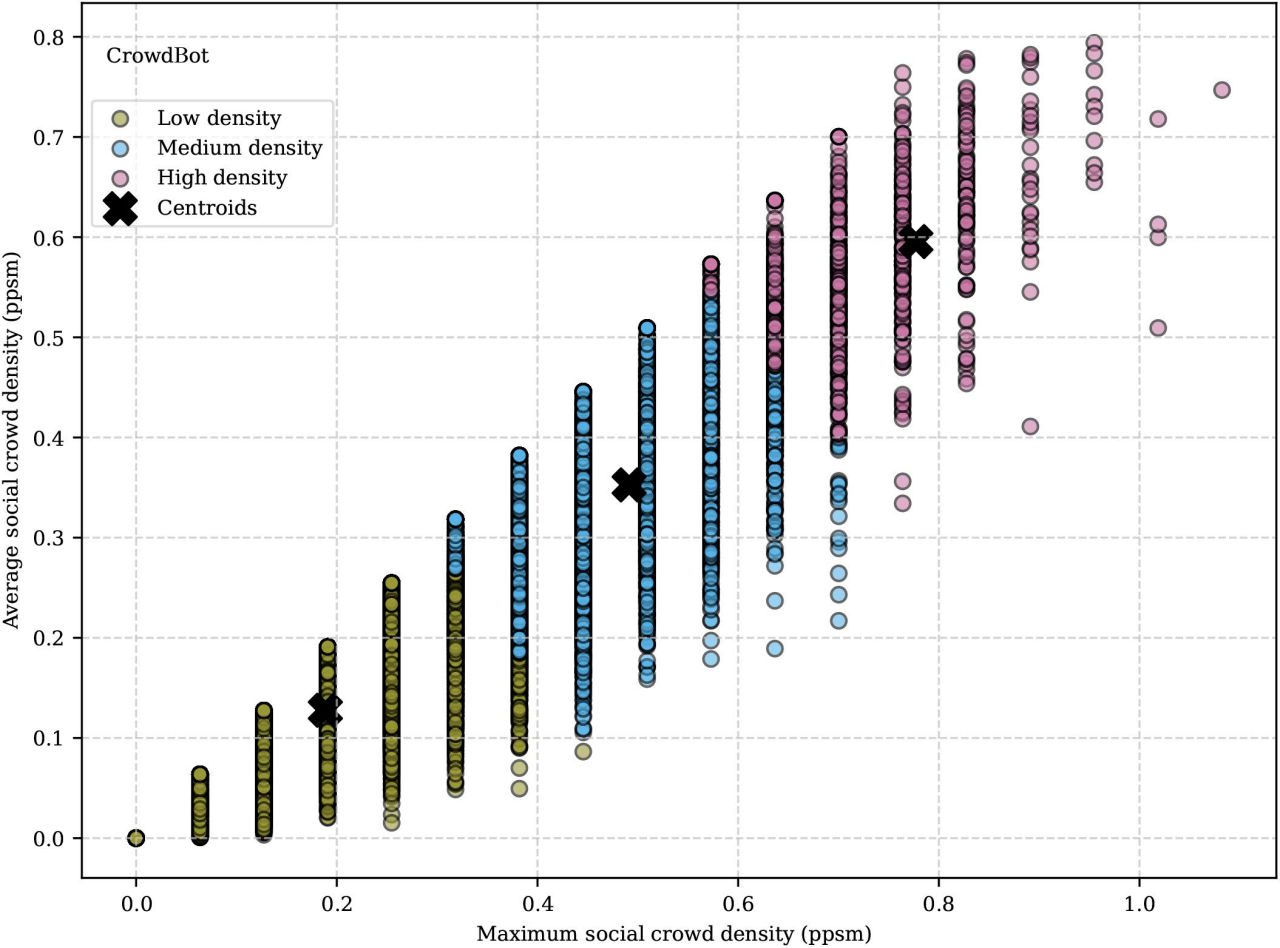
Stratification of pedestrian tracks based on local crowd density. Pedestrian trajectories from the CrowdBot dataset were clustered using weighted *k*-means++ into low-, medium-, and high-density categories. Clustering was performed in a 2D feature space defined by the maximum and average crowd density experienced during each trajectory.

The final clustering produced the following centroid densities in ppsm: low density (0.19, 0.10), medium density (0.49, 0.28), and high density (0.78, 0.50), where each pair denotes the maximum and average local density, respectively. The resulting stratification is illustrated in Fig. 9.

### Minimum distance computation

All distances were computed as edge-to-edge separations over common frames of valid agent-pedestrian pairs.

#### Pairs, coappearance, and distance trace

Let *i* denote an agent, either a pedestrian or the robot, and let *j* denote a pedestrian distinct from *i*. For pedestrian-centered analyses, pairs (*i*, *j*) are pedestrian-pedestrian; for robot-centered analyses, pairs are robot-pedestrian. Let *T_ij_* be the set of frames in which *i* and *j* coappear, and let

$$d_{\mathrm{ij}}\left(t\right),\;t\in T_{\mathrm{ij}}$$ (2)

denote their edge-to-edge distance time series.

#### Comotion exclusion

To reduce the influence of within-group proxemics without inferring true social-group membership from trajectories, we applied a comotion exclusion based on persistent comovement. For any pair (*i*, *j*), define the distance excursion

$$\Delta d_{\mathrm{ij}}=\underset{t\in T_{\mathrm{ij}}}{\text{max}}\;d_{\mathrm{ij}}\left(t\right)-\underset{t\in T_{\mathrm{ij}}}{\text{min}\;}d_{\mathrm{ij}}\left(t\right)$$ (3)

A pair is excluded if

$$|T_{\mathrm{ij}}|\cdot \Delta t>1\;s\;\text{and}\;\Delta d_{\mathrm{ij}}\leq \Delta d_{\text{th}}$$ (4)

with $\Delta d_{\text{th}}=2\;m$, where Δ*t* is the frame period. This rule removes persistently comoving pairs that likely correspond to within-group motion while accepting that it also excludes some nongroup cases such as sustained following. To minimize differential bias across conditions, the same exclusion was applied to both pedestrian-pedestrian and robot-pedestrian pairs. To assess the dependence of the proxemics results on this exclusion rule, we performed a sensitivity analysis over $\Delta d_{\text{th}}$ and report the results in the Supplementary Materials. It shows that, without comotion exclusion, within-group proxemics dominate the observed effects, whereas, at $\Delta d_{\text{th}}=2\;m$, most of these effects are removed and the results are stable around this parameter choice.

#### Per-pair minimum and near-minimum band

For any valid pair (*i*, *j*), define the per-pair minimum distance

$$d_{\mathrm{ij}}^{\text{min}}=\underset{t\in T_{\mathrm{ij}}}{\text{min}}\;d_{\mathrm{ij}}\left(t\right)$$ (5)

and the near-minimum band with width δ=0.2 m

$$B_{\mathrm{ij}}=\left\{t\in T_{\mathrm{ij}}:d_{\mathrm{ij}}\left(t\right)\leq d_{\mathrm{ij}}^{\text{min}}+\delta \right\}$$ (6)

#### Per-agent instantaneous minimum and aggregate distribution

For agent *i* at time *t*, let

$$N_{i}\left(t\right)=\left\{j:t\in B_{\mathrm{ij}}\right\}$$ (7)

be the set of partners whose pair is in a near-minimum state at *t*. The instantaneous minimum distance for agent *i* is then

$$d_{i}^{\text{min}}\left(t\right)=\left\{\begin{array}{cc} \underset{j\in N_{i}\left(t\right)}{\text{min}}d_{\mathrm{ij}}\left(t\right), & N_{i}\left(t\right)\neq \emptyset \\ \text{undefined}, & N_{i}\left(t\right)=\emptyset \end{array}\right.$$ (8)

Thus, $d_{i}^{\text{min}}\left(t\right)$ is defined only on frames where at least one pair involving *i* lies within 0.2 m of its per-pair minimum. Frames without any such partner are omitted for that agent. Aggregating these $d_{i}^{\text{min}}\left(t\right)$ values across agents yields the reported minimum-distance distributions.

#### Kernel density estimation (KDE)

Minimum-distance densities were estimated using Gaussian kernel density estimation. The bandwidth followed Silverman’s rule with a robust scale

$$h_{0}=0.9\;\sigma_{r}\;n^{-1/5},\;\sigma_{r}=\text{min}\;\left(s,\;\frac{\text{IQR}}{1.349}\right)$$ (9)

where *n* is the sample size, *s* is the sample SD, and IQR is the interquartile range. To harmonize smoothness across datasets, we applied dataset-specific scaling $h=\kappa_{d}\;h_{0}$ with $\kappa_{\text{CrowdBot}}=\kappa_{\text{SCAND}}=1.0$ and $\kappa_{\text{JRDB}}=\kappa_{\text{SiT}}=0.65$.

#### Close-pass regime and maximum excess clearance

To summarize the separation between robot-centered and pedestrian-centered minimum-distance distributions, we compared the kernel density estimates of the Robot and HRI-ped groups. Let ${\hat{f}}_{\text{HRI}}\left(d\right)$ denote the HRI-ped density and ${\hat{f}}_{\text{Robot}}\left(d\right)$ the Robot density. We first identified the first prominent mode of ${\hat{f}}_{\text{Robot}}\left(d\right)$ for d≥0. Starting from this mode and scanning toward smaller distances, we then located the first crossing between ${\hat{f}}_{\text{Robot}}\left(d\right)$ and ${\hat{f}}_{\text{HRI}}\left(d\right)$; this crossing defined the equalization distance $d_{\text{eq}}$. The close-pass regime was defined as the interval $d\leq d_{\text{eq}}$.

Within this regime, we compared the left branches of the two KDEs across a dense set of iso-density levels. For each density level *y*, we determined the smallest distance $d_{\text{Robot}}\left(y\right)$ and $d_{\text{HRI}}\left(y\right)$ at which the corresponding KDE reached that level, provided both distances lay within the close-pass regime. The maximum excess clearance Δ was then defined as the largest signed horizontal gap

$$\Delta =\underset{y}{\text{max}}\left[d_{\text{Robot}}\left(y\right)-d_{\text{HRI}}\left(y\right)\right]$$ (10)

over the scanned iso-density levels within the close-pass regime. Positive Δ indicates that the Robot distribution is shifted toward larger clearances than the HRI-ped distribution. Thus, Δ quantifies the maximum robot-specific clearance shift in meters, whereas $d_{\text{eq}}$ determines the extent of the close-pass regime over which this separation remains visible.

### Additional filtering and quality control

Several additional preprocessing steps were incorporated into the pipeline to improve data reliability and accuracy.

1) To compute minimum distance and crowd density, we decreased the requirement for the track length of 3.5 s and included pedestrians with track lengths of at least 1 s (ground-truth annotations) or 1.5 s (model-based annotations).

2) To minimize the influence of odometry error on pedestrian position data and consequently calculated motion metrics, we removed frames from the datasets that corresponded to fast, significant turns of the navigating robot.

3) Significant position noise from imperfect odometry was sometimes observed when pedestrians were actually stationary or moving very slowly, leading to significant velocity, acceleration, and jerk values. We detected the affected frames and set their velocity to a constant of 0 m s^−1^ before deriving the finite difference motion metrics.

4) In the JRDB dataset specifically, spurious odometry noise occasionally introduced position spikes lasting up to 5 frames (0.35 s), leading to artificially high jerk values. To eliminate these artifacts, we identified these occurrences and interpolated the positions between the affected regions.

5) Crowd density and minimum distance calculations for the CrowdBot, SCAND, and SiT datasets were computed using frames where the robot’s velocity exceeded 0.4 m s^−1^. This was to ensure a fair comparison, as for pedestrians, only moving frames were considered. In the JRDB, excluding stationary robot frames was not feasible due to the substantial proportion of such instances relative to the whole dataset size.

### Evaluation metrics derivation

We defined motion-based and proximity-based metrics to evaluate pedestrian behavior in the presence of robots. All comparisons were conducted separately for each dataset.

#### Motion dynamics metrics

We analyzed angular velocity, linear acceleration, and linear jerk as complementary descriptors of pedestrian adaptation. Under unobstructed conditions, pedestrians tend to move at constant speed and steady heading toward their goals. Accordingly, angular velocity captures changes in heading, linear acceleration captures deviations from constant walking speed, and linear jerk captures the abruptness of those speed changes.

##### Individual behavioral difference

For pedestrians with at least 1.5 s of data in both interacting and noninteracting subsets, we computed normalized differences in average absolute angular velocity, linear acceleration, and linear jerk between the two contexts. Normalization by each pedestrian’s trajectory-wide mean reduced the influence of intrinsic behavioral variability and emphasized interaction-related changes.

##### Significant motion events

We analyzed significant events, defined as interactions in which either the turn angle exceeded 45° or the velocity change exceeded 2 km hour^−1^ within 1 s. These events represent comparatively sudden turning or speed adjustment, where pedestrians likely had less opportunity to rely on gradual anticipatory adaptation.

#### Proximity metrics

We evaluated proximity using minimum distance and comfort-zone intrusions. Both metrics were computed in an edge-to-edge manner, accounting for the physical dimensions of each agent. Pedestrians were modeled as circles with radius 0.25 m, whereas robots were represented by their minimum bounding rectangles.

##### Minimum distance

Minimum distance captures the smallest edge-to-edge separation between an agent and nearby pedestrians in the vicinity of closest approach. For each valid agent-pedestrian pair, we computed the per-pair minimum distance and defined a near-minimum band consisting of frames within 0.2 m of that minimum. At each such frame, we recorded the smallest separation to any concurrent partner, yielding a per-agent time series concentrated on close-pass interactions. To reduce bias from within-group proxemics, we applied the comotion exclusion defined above to both pedestrian-pedestrian and robot-pedestrian pairs. The resulting minimum-distance distributions are summarized using the close-pass regime and the maximum excess clearance Δ.

##### Intrusion count

Intrusion count quantifies how many pedestrians enter a 0.3-m edge-to-edge comfort zone around the reference agent. We summarized these intrusions as relative frequencies and as ratios of mean intrusion counts between pedestrian-centered and robot-centered conditions. The 0.3-m threshold was grounded in anchors reported for everyday avoidance, where typical passing distances are about 0.75 to 0.8 m center to center (*6*).

##### Spacing-speed relationship

To evaluate how pedestrian clearance varies with speed, we analyzed the dependence of minimum edge-to-edge distance on instantaneous robot velocity and, as a control, on pedestrian velocity. This analysis was performed within each density stratum for CrowdBot, the only dataset with sufficient robot-speed variation for a robust density-stratified comparison. We fitted locally weighted scatterplot smoothing curves to these relationships to capture general trends without assuming a specific functional form. Smoothing was applied with a span of 0.3 and two robustifying iterations to reduce the influence of outliers.

### Statistical significance analysis

To evaluate statistical differences between HRI-ped and HHI-ped interaction groups, we applied nonparametric tests. All analyses were performed on per-pedestrian statistics to ensure independent and identically distributed samples.

For the local motion dynamics comparison (Fig. 4), statistical significance between HRI-ped and HHI-ped distributions was assessed using a two-sided Mann-Whitney *U* (MWU) test. To obtain one independent sample per pedestrian, we first computed the time-averaged absolute values of each metric for all frames belonging to a given individual and then took the mean of those values. The resulting per-pedestrian means formed the distributions compared with the MWU test.

For the individual-level motion characteristic analysis (Fig. 5), the same two-sided MWU test was used. In this case, each data point already represented a single pedestrian’s normalized change between interacting and noninteracting segments, so the MWU was applied directly to these per-subject distributions.

For the significant behavioral event analysis, statistical differences between HRI-ped and HHI-ped were evaluated using a two-sample multivariate energy-distance test. The test was applied to paired features (velocity change and turn angle), which were standardized before computation. The resulting *P* values reflect differences in the joint multivariate distribution of these behavioral event descriptors.

For group-level motion-dynamics comparisons, effect sizes were reported as Wasserstein-1 distances in the physical units of each metric. For individualized normalized-change comparisons, Cohen’s *d* was reported because the compared quantities are dimensionless baseline-relative changes.
