# Supplementary material for: Assessing pedestrian responses to autonomous and personal mobility robots in crowded public spaces
Source: Sci Adv. 2026 Aug 5;12(32):eaef2576. doi: 10.1126/sciadv.aef2576 (PMC13440412; doi:10.1126/sciadv.aef2576)
Supplement: Supplementary file 1 [file sciadv.aef2576_sm.pdf]

Science Advances
AAAS

Supplementary Materials for

Assessing pedestrian responses to autonomous and personal mobility robots in crowded public spaces

Dominik Wojcikiewicz *et al*.

Corresponding author: Dominik Wojcikiewicz, dominik.wojcikiewicz@hest.ethz.ch;
Diego Paez-Granados, diego.paez@hest.ethz.ch

Sci. Adv. **12**, eaef2576 (2026)
DOI: 10.1126/sciadv.aef2576

The PDF file includes:

Supplementary Text
Tables S1 to S3
Figs. S1 to S11
Legend for movie S1

Other Supplementary Material for this manuscript includes the following:

Movie S1

Supplementary Text

Dataset technical details

Table S1: **Technical characteristics of the datasets used in this study.** Frames used count reflects data retained after filtering.

**Attribute**	**CrowdBot**	**SCAND**	**JRDB**	**SiT**
Robot Type	PMV (Qolo)	UGV (Jackal/Spot)	UGV (JackRabbot)	UGV (Husky)
Frames	253,332	311,661	54,913	9,801
Frames used	218,701	292,475	37,621	1,994
LiDAR Freq. (Hz)	19.58	9.93	14.50	10.05
LiDAR	2×VLP-16	1×VLP-16	2×VLP-16	2×VLP-16
Cameras	1×D435	Kinect RGB-D, stereo/mono	1×OmniStereo	5×Basler a2A1920
GT Labels	No	No	Yes	Yes

Robot and pedestrian speed regimes across datasets

The four datasets differ substantially in their robot-speed operating regimes, which is important for interpreting the cross-dataset proxemics results in the main text. Figure S1 shows that CrowdBot spans a broad range of robot velocities, SCAND concentrates on a narrower but higher-speed regime, JRDB is dominated by near-stationary robot motion, and SiT occupies a narrower intermediate-speed regime. This distributional structure is one reason CrowdBot is the only dataset suitable for the density-stratified within-dataset speed-dependence analysis in the main text.

Pedestrian-speed distributions are broad in all four datasets (Figure S2), but their dominant walking-speed ranges differ across datasets.

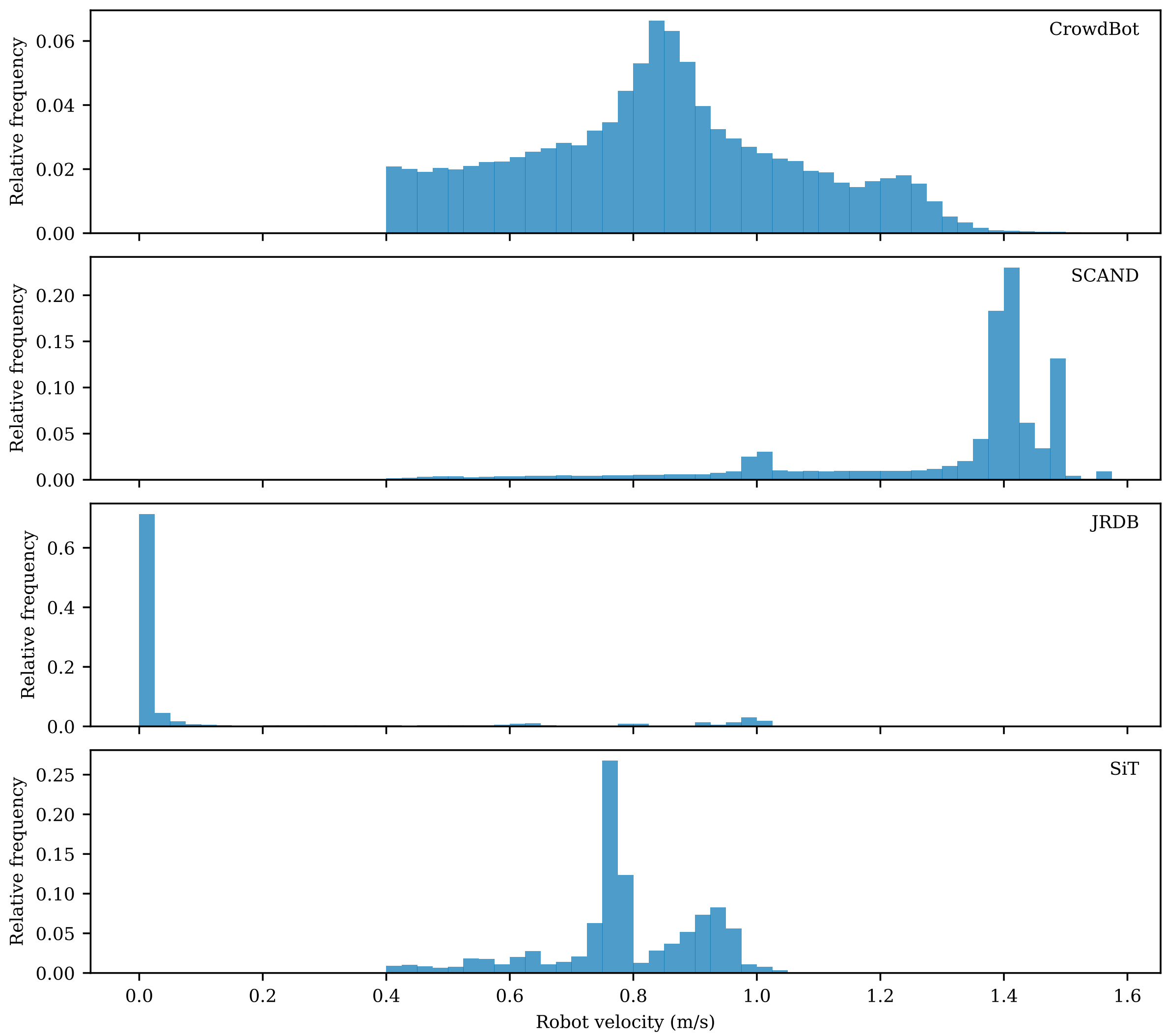

Figure S1: **Robot velocity distributions across datasets.** CrowdBot spans the broadest range of robot velocities, SCAND is concentrated in a narrower but higher-speed regime, JRDB is dominated by near-stationary robot motion, and SiT occupies a narrower intermediate-speed regime. These differences support the cross-dataset interpretation of proxemics reported in the main text.

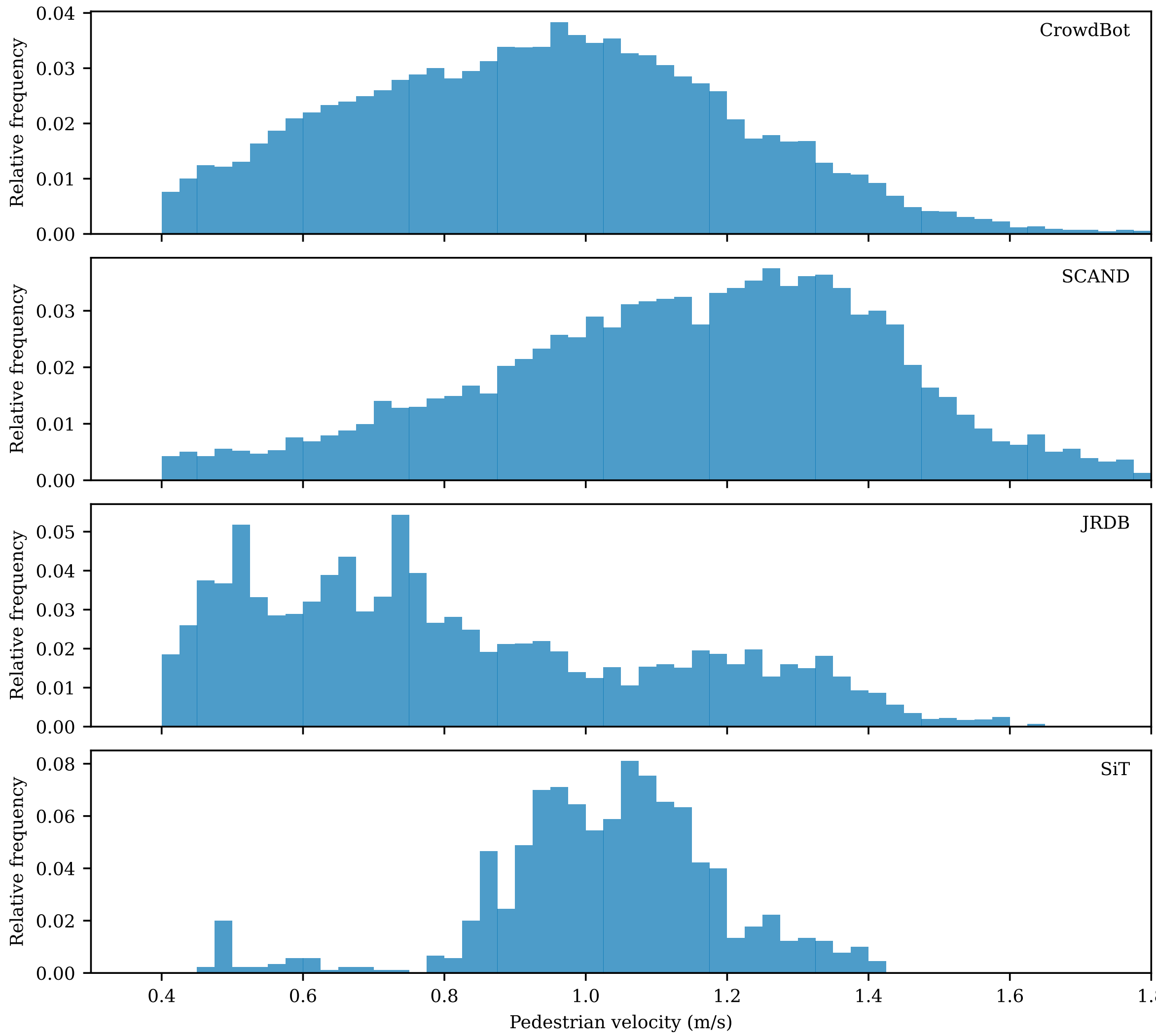

Figure S2: **Pedestrian velocity distributions across datasets.** Pedestrian walking speeds are broadly distributed in all four datasets but differ in their dominant ranges.

Validation of model-based motion metrics

To assess whether model-based tracking introduces bias into our analysis, we compared **model-generated tracks** against **ground-truth annotations** in the JRDB dataset. Rather than focusing on the absolute values of motion metrics for each group, we evaluated whether the **relative behavioral differences** between NI-ped and HRI-ped were preserved across tracking methods, since this contrast forms the core analytical quantity in our study.

Specifically, we computed mean angular velocity, linear acceleration, and linear jerk for each group and examined the density-averaged difference in sample means, $\bar{x}_{\text{NI-ped}} - \bar{x}_{\text{HRI-ped}}$. These contrasts were then compared between tracks obtained from ground-truth annotations and those generated by our tracking pipeline (Figure S3).

The results show strong correspondence between predicted and annotated contrasts, with only minor deviations across metrics. This indicates that model-based tracking does not introduce a systematic bias into the interaction-related behavioral differences used in the main analyses.

Accordingly, we used ground-truth annotations for JRDB and SiT, and model-based trajectories for CrowdBot and SCAND, where ground-truth pedestrian trajectories were not available.

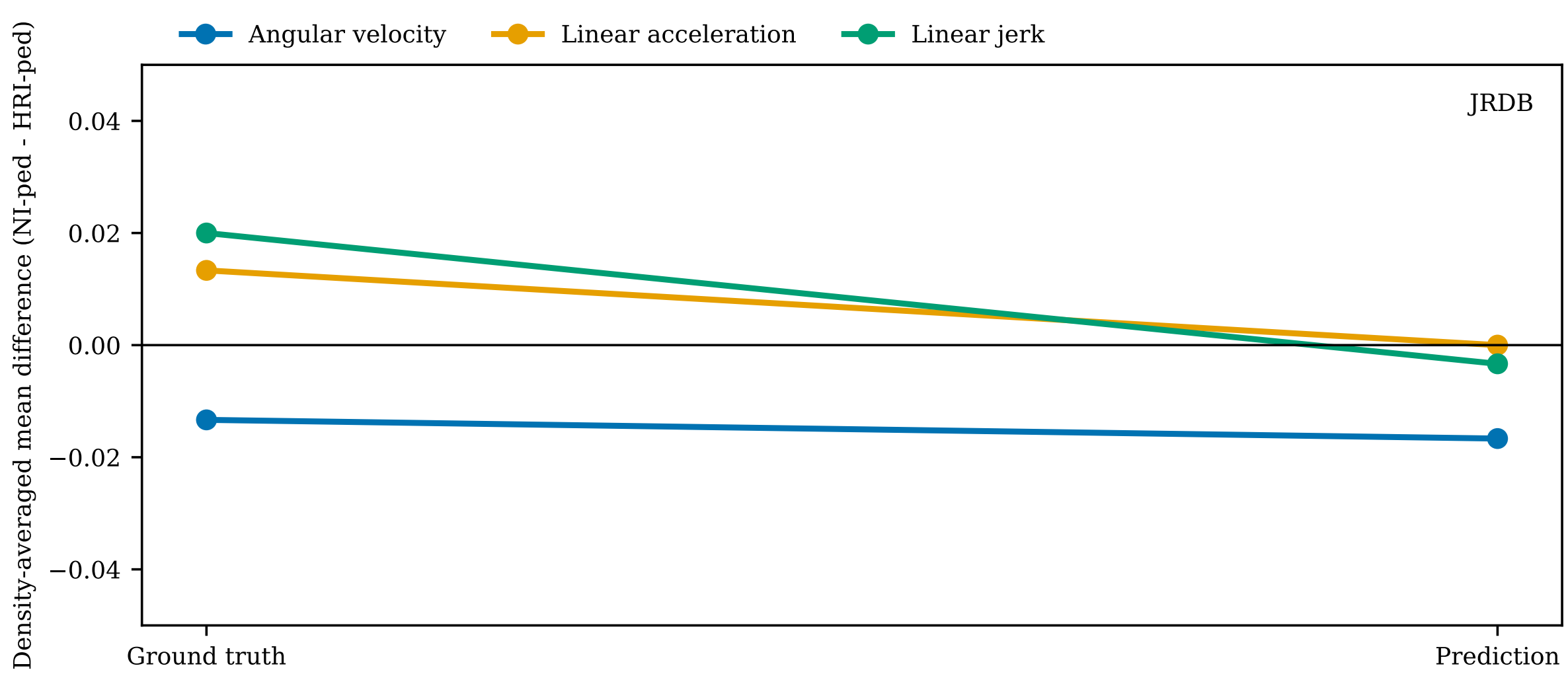

Figure S3: **Validation of model-based tracking against ground truth in JRDB.** Density-averaged mean differences in angular velocity (ω), linear acceleration (a), and linear jerk (j) between NI-ped and HRI-ped are shown for ground-truth and generated trajectories. Their close agreement indicates that the interaction-related contrasts in the main analyses are preserved under model-based tracking.

Sensitivity analysis for interaction labeling

To assess the robustness of the frame-wise interaction-labeling rule, we varied the key parameters of the designated-agent interaction definition from more direct to more anticipatory settings. The default parameters used in the main analysis were:

(i) Angular threshold limits: $\alpha_{dyn}^{\min} = 35°$, $\alpha_{dyn}^{\max} = 100°$.

(ii) Edge-to-edge perception distance limits: $d_{\alpha_{dyn}}^{\min} = 0.5$ m, $d_{\alpha_{dyn}}^{\max} = 3.0$ m.

(iii) Time threshold for ttcc: $\text{ttcc}_{\text{thresh}} = 3.0$ s.

(iv) Collision threshold for ttcc: $\text{ttcc}_{d} = 0.8$ m.

We evaluated six additional variants, three increasingly direct and three increasingly anticipatory, whose parameters are listed in Table S2. The analysis was performed on CrowdBot, which provides the largest number of interaction samples and the broadest behavioral coverage.

For each parameter variant, we computed the normalized per-pedestrian motion changes between interacting and non-interacting segments for both HRI-ped and HHI-ped (Figure S4). Across both interaction settings, moving from the more anticipatory variants toward the default and then more direct variants produced modest but systematic increases in the normalized motion changes. This pattern is consistent with stricter interaction definitions placing greater emphasis on closer, shorter-horizon encounters, in which turning and speed adjustments are more pronounced, while looser anticipatory variants include a broader range of earlier and weaker interaction frames. At the same time, the increase remained gradual rather than abrupt over the central range of parameter choices, indicating that the inferred motion differences are not highly sensitive to the exact setting near the adopted default. We therefore interpret the main conclusions as robust to moderate variation in the interaction-labeling parameters, while noting that increasingly direct variants shift the analysis toward more reactive and higher-magnitude encounters.

Table S2: **Parameter sets for the interaction-labeling sensitivity analysis.**

**Variant**	$\alpha_{dyn}^{\min}$	$\alpha_{dyn}^{\max}$	$d_{\alpha_{dyn}}^{\min}$	$d_{\alpha_{dyn}}^{\max}$	$\mathrm{ttcc}_{\mathrm{thresh}}$	ttcc_d
D3	25°	80°	0.5 m	2.0 m	1.5 s	0.5 m
D2	30°	85°	0.5 m	2.5 m	2.0 s	0.6 m
D1	30°	85°	0.5 m	2.5 m	2.5 s	0.7 m
Default	35°	100°	0.5 m	3.0 m	3.0 s	0.8 m
A1	40°	110°	0.5 m	3.5 m	3.5 s	0.9 m
A2	40°	110°	0.5 m	3.5 m	4.0 s	1.0 m
A3	45°	115°	0.5 m	4.0 m	4.5 s	1.1 m

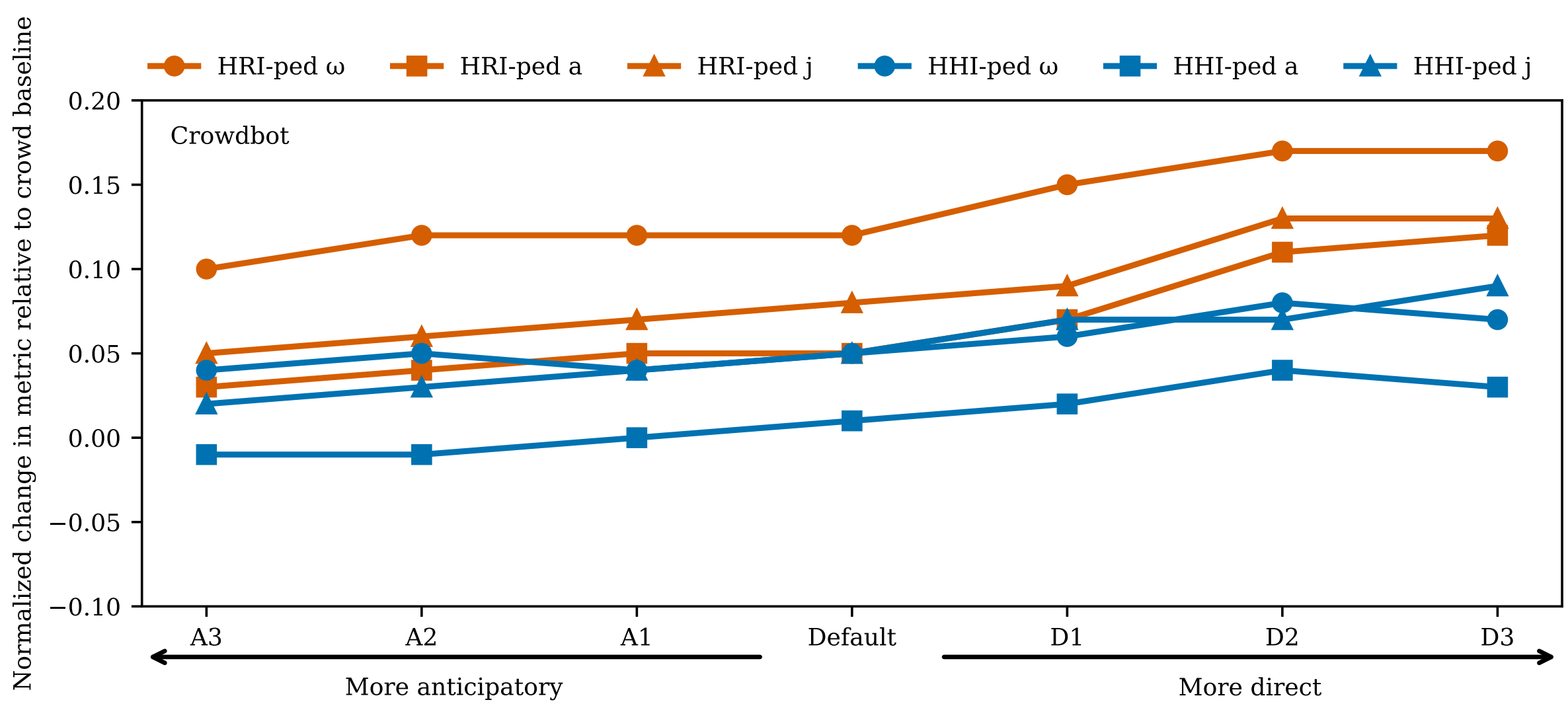

Figure S4: **Sensitivity of the interaction-labeling rule to parameter variation.** Normalized per-pedestrian motion changes are shown for HRI-ped and HHI-ped across increasingly anticipatory (A3–A1), Default, and increasingly direct (D1–D3) parameter variants. HRI-ped changes increase gradually under stricter, more direct criteria, whereas HHI-ped changes vary less while showing the same trend. The stable central range around Default supports the parameter choice used in the main analyses.

Sensitivity analysis for motion processing

To assess the dependence of the motion-dynamics analyses on the choice of motion-processing parameters, we repeated the CrowdBot group-level motion analysis across a family of progressively lighter and heavier motion processing settings. In each variant, we varied the position-smoothing window together with the finite differences used to derive linear velocity, linear acceleration, linear jerk, and turn angle. All tested variants are listed in Table S3.

Figure S5 shows that the absolute magnitudes of angular velocity, linear acceleration, and linear jerk decrease monotonically as the processing becomes heavier, as expected from increased temporal averaging. Lighter variants preserve more short-horizon variation and therefore yield larger absolute values, whereas heavier variants smooth more strongly and compress the metric magnitudes.

However, the main conclusion of the motion analysis is not driven by this scaling effect. Figure S6 shows that the Wasserstein distances between HRI-ped and HHI-ped remain small across the full range of processing variants and preserve the same qualitative pattern as in the main text. Thus, although the absolute motion-metric magnitudes depend on the chosen smoothing and finite difference windows, the conclusion that HRI-ped and HHI-ped motion-dynamics distributions remain closely matched is robust to these parameter choices. We selected the configuration used in the main analyses because a 1.0 s window corresponds approximately to two human steps and therefore captured ordinary short-horizon walking adjustments at a physiologically interpretable scale. For jerk, we used a shorter 0.5 s window to retain sensitivity to more abrupt adjustments.

Table S3: **Motion-processing variants used in the sensitivity analysis.** Variant names follow the velocity/acceleration/turn finite difference window shown in the figures, with the approximate equivalent number of human steps in parentheses. The bold row indicates the configuration used in the main analyses.

Variant	Pos. smooth window	Vel. window	Acc. window	Jerk window	Turn window
0.50 s (1 step)	1.00 s	0.50 s	0.50 s	0.33 s	0.50 s
0.75 s (1.5 steps)	1.25 s	0.75 s	0.75 s	0.40 s	0.75 s
0.88 s (1.75 steps)	1.38 s	0.875 s	0.875 s	0.45 s	0.875 s
**1.00 s (2 steps)**	**1.50 s**	**1.00 s**	**1.00 s**	**0.50 s**	**1.00 s**
1.13 s (2.25 steps)	1.75 s	1.125 s	1.125 s	0.625 s	1.125 s
1.25 s (2.5 steps)	2.00 s	1.25 s	1.25 s	0.75 s	1.25 s
1.50 s (3 steps)	2.50 s	1.50 s	1.50 s	1.00 s	1.50 s

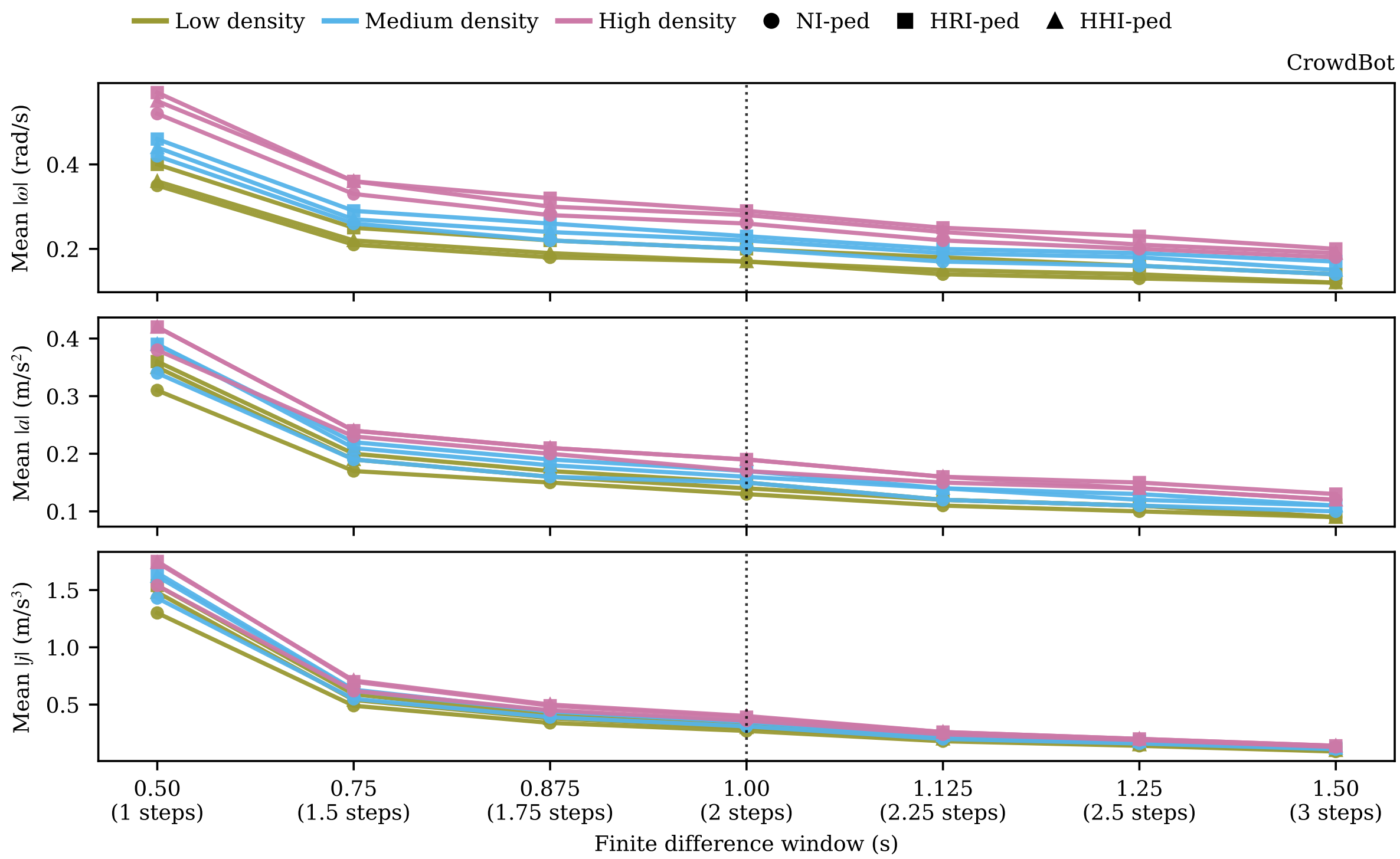

Figure S5: **Sensitivity of absolute motion-metric magnitudes to the motion-processing variant in CrowdBot.** Mean absolute angular velocity, linear acceleration, and linear jerk are shown for NI-ped, HRI-ped, and HHI-ped across progressively lighter and heavier processing variants. As expected, heavier variants reduce the absolute metric magnitudes through stronger temporal averaging. The dotted vertical line marks the 1.00 s (2 steps) configuration used in the main analyses.

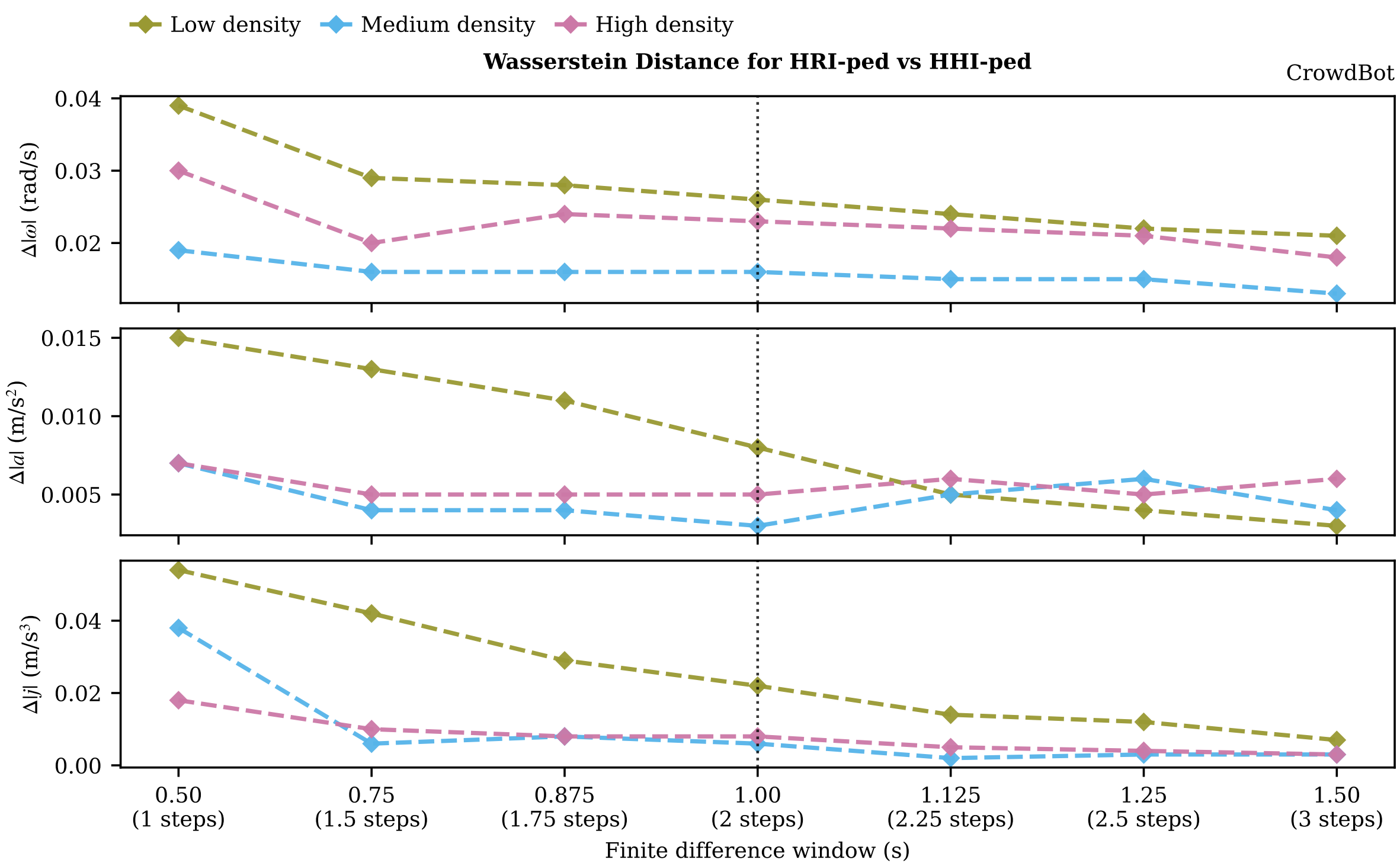

Figure S6: **Sensitivity of HRI-ped versus HHI-ped motion differences to the motion-processing variant in CrowdBot.** Wasserstein distances between HRI-ped and HHI-ped are shown for angular velocity, linear acceleration, and linear jerk across progressively lighter and heavier processing variants. The distribution-level differences remain small across the full range, indicating that the main motion-dynamics conclusion is robust to the motion-processing choice.

Sensitivity analysis for co-motion exclusion of within-group proxemics

A substantial fraction of pedestrians in public spaces travel in groups and therefore maintain systematically smaller interpersonal distances than unrelated pedestrians. If such within-group proxemics are retained, they can bias the pedestrian-centered proxemics baselines and obscure the robot-specific spacing effects of interest. Because inferring true social-group membership from trajectories alone is challenging, we did not attempt to solve the group-detection problem directly. Instead, we applied a co-motion exclusion based on persistent co-movement, which removes many likely within-group pairs while also accepting that some non-group cases, such as sustained following, are excluded as well. To minimize any differential bias between conditions, the same exclusion was applied to both pedestrian–pedestrian and robot–pedestrian pairs.

The co-motion threshold Δd_{th} controls how much a pair's edge-to-edge distance is allowed to vary while still being treated as persistently co-moving and therefore excluded. Small values exclude only pairs whose distance remains nearly constant, whereas larger values progressively exclude a broader set of co-moving pairs, including more following-like interactions. To assess the role of this exclusion, we varied Δd_{th} over a wide range in CrowdBot. Here, $\Delta d_{\text{th}} = 0$ corresponds to retaining all proxemics interactions, whereas larger values progressively exclude more persistently co-moving pairs.

As a summary quantity, we used the ratio of mean comfort-zone intrusion counts,

$$\frac{\mathbb{E}[\text{HRI-ped}]}{\mathbb{E}[\text{Robot}]},$$

which is reported in the main-text intrusion analysis (Figure 7). Values larger than 1 indicate that pedestrians in the HRI-ped subset experience more comfort-zone intrusions than the robot, that is, the robot's comfort zone is invaded less often than the corresponding pedestrian-centered baseline.

Figure S7 shows that when $\Delta d_{\text{th}} = 0$, the ratio is very large, especially in low density, indicating that retained co-moving pedestrian pairs strongly inflate the pedestrian-centered intrusion counts. As Δd_{th} increases, the ratio drops sharply and then stabilizes near the value used in the main analysis, $\Delta d_{\text{th}} = 2$ m. This pattern indicates that the exclusion is working as intended: it removes the dominant within-group proxemics effect while preserving stable robot-versus-pedestrian proxemics differences over the parameter range around the adopted threshold. We therefore chose $\Delta d_{\text{th}} = 2$ m as a conservative operating point at which most of the within-group proxemics effect has already been

removed, while limiting the additional exclusion of non-group cases such as sustained following with distance changes below 2 m.

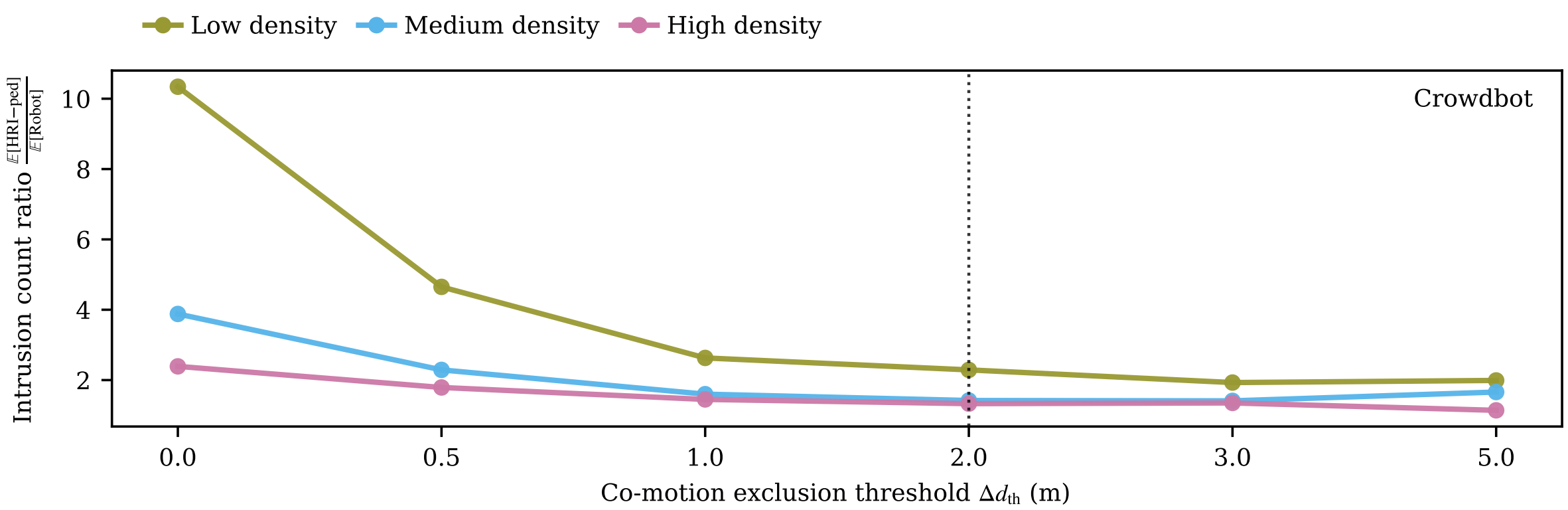

Figure S7: **Sensitivity of the intrusion-count ratio to the co-motion exclusion threshold in CrowdBot.** The plotted quantity is the ratio of mean comfort-zone intrusion counts, $\mathbb{E}[\text{HRI-ped}]/\mathbb{E}[\text{Robot}]$, for low-, medium-, and high-density conditions as a function of the co-motion threshold Δd_{th}. At $\Delta d_{\text{th}} = 0$, all proxemics interactions are retained and within-group proxemics strongly inflate the pedestrian-centered baseline, especially in low density. As Δd_{th} increases, the ratios drop sharply and stabilize near the adopted threshold, $\Delta d_{\text{th}} = 2$ m, supporting the robustness of the main proxemics conclusions.

Supplementary cross-dataset motion results

This section reports the motion-related results for SCAND, JRDB, and SiT that are not shown in the main text. These results complement the CrowdBot findings and provide supporting evidence across a larger and more diverse set of datasets.

Motion dynamics similarity across datasets. SCAND, JRDB, and SiT all showed the same overall pattern as CrowdBot: the distributions of angular velocity, linear acceleration, and linear jerk remained closely matched between HRI-ped and HHI-ped (Figure S8). SCAND provides additional large-scale evidence in a higher-speed robot regime. JRDB and SiT are smaller, but use ground-truth pedestrian annotations and likewise do not indicate a clear or conclusive HRI-ped versus HHI-ped difference. In SiT, only the lower-density strata contained sufficient data for comparison.

Individualized motion changes across datasets. The individualized analysis shows the same qualitative conclusion across SCAND, JRDB, and SiT (Figure S9). SCAND again shows closely matched HRI-ped and HHI-ped distributions. JRDB and especially SiT exhibit greater variability due to smaller sample sizes, but do not indicate a consistent individualized-motion advantage for either interaction type.

Significant motion events across datasets. The significant-event analysis also supports the same qualitative conclusion (Figure S10). Across SCAND, JRDB, and SiT, the HRI-ped and HHI-ped event maps in the joint space of velocity change over 1 s and turn angle over 1 s remain similar, and the corresponding multivariate comparisons do not indicate a significant difference. The smaller datasets, especially SiT, are visibly limited by low event counts.

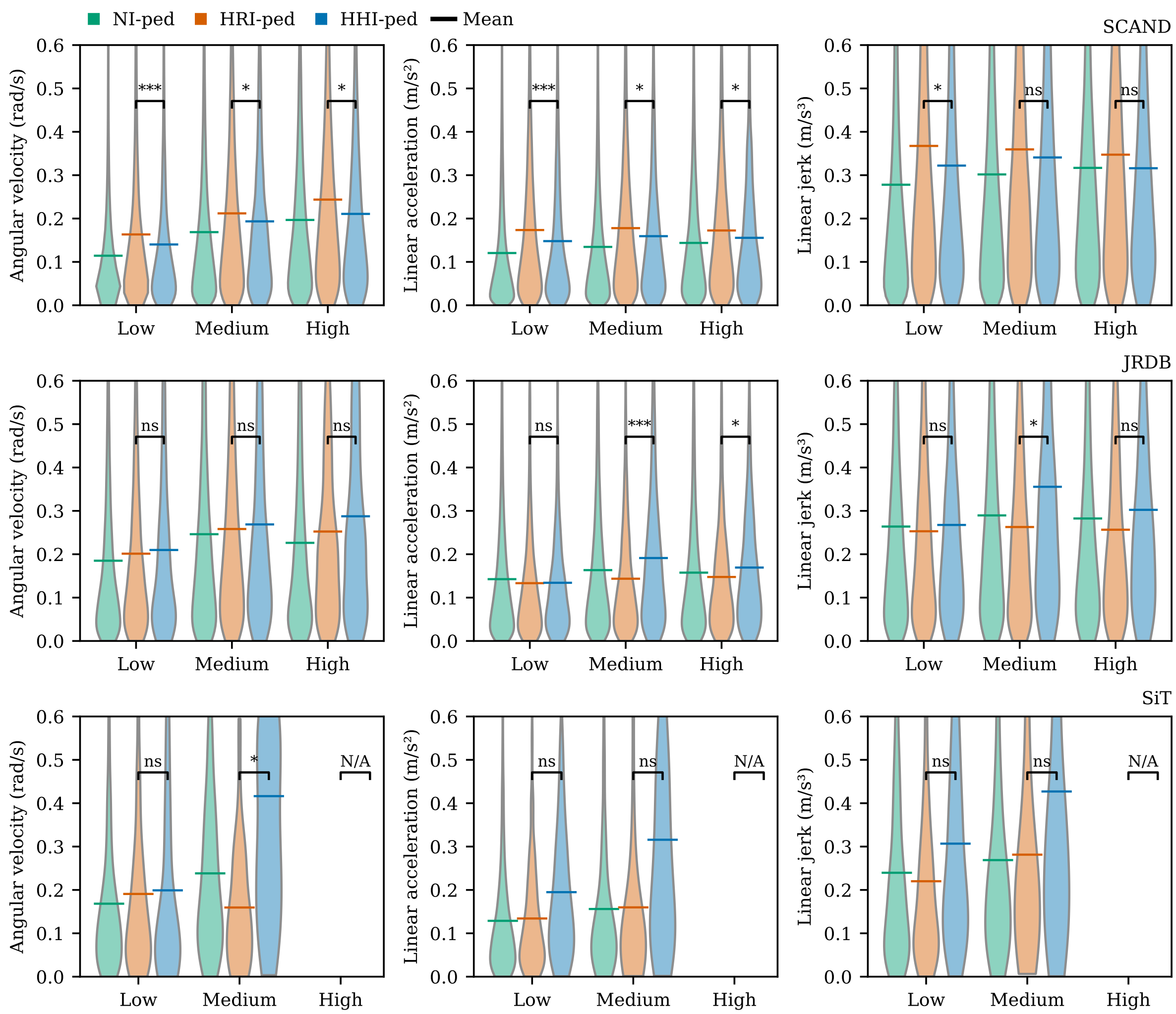

Figure S8: **Supplementary motion-dynamics comparisons for SCAND, JRDB, and SiT.** Violin plots show angular velocity, linear acceleration, and linear jerk for NI-ped, HRI-ped, and HHI-ped across available density strata. As in CrowdBot, HRI-ped and HHI-ped remain closely matched across metrics and densities, with sample-size limitations most apparent in SiT.

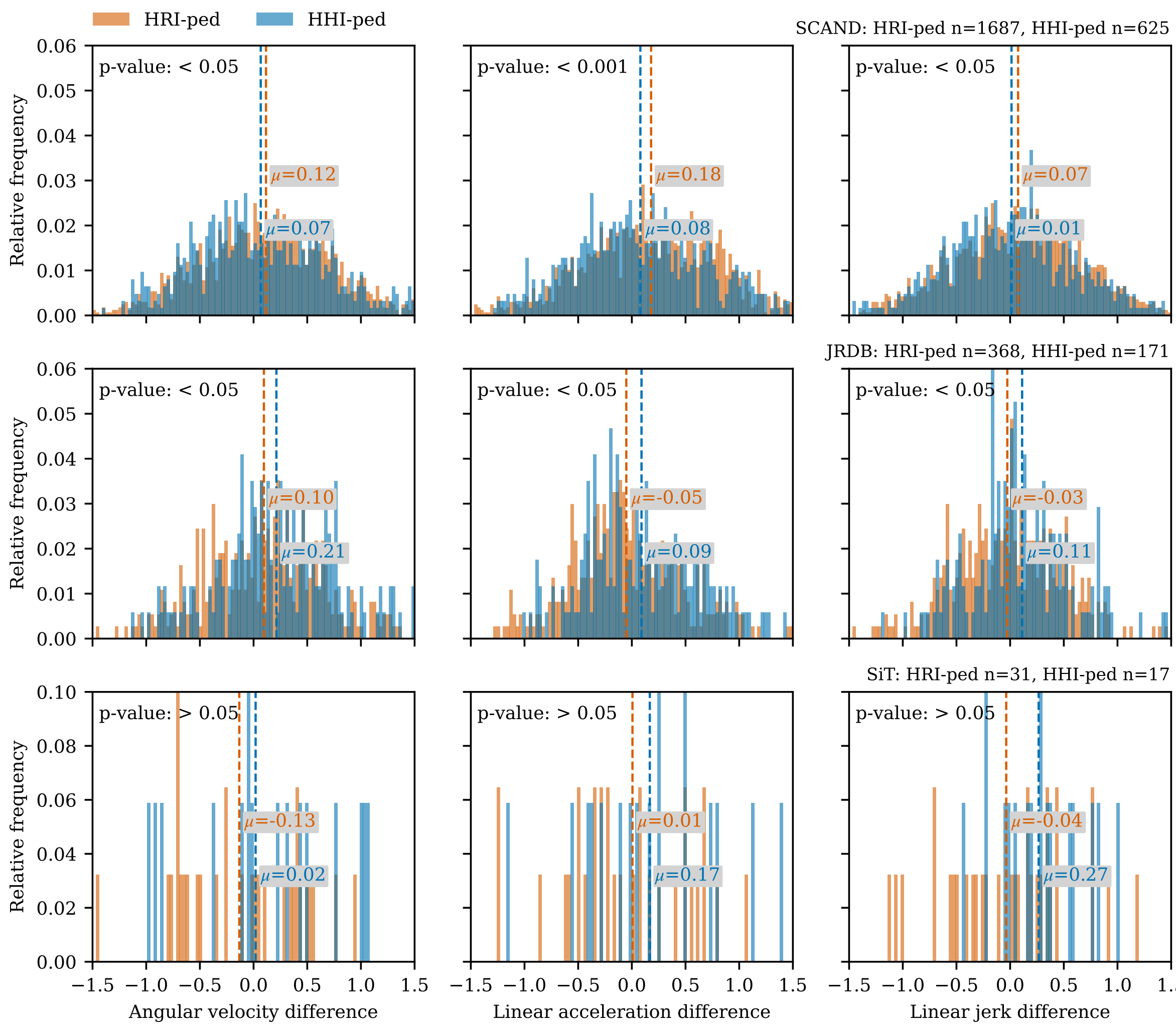

Figure S9: **Supplementary individualized motion changes for SCAND, JRDB, and SiT.** Normalized per-pedestrian motion changes relative to trajectory-wide behavior are shown for HRI-ped and HHI-ped. SCAND provides additional large-scale supporting evidence, whereas JRDB and SiT show greater variability because of smaller sample sizes. Across datasets, no clear or consistent HRI-ped versus HHI-ped difference is observed.

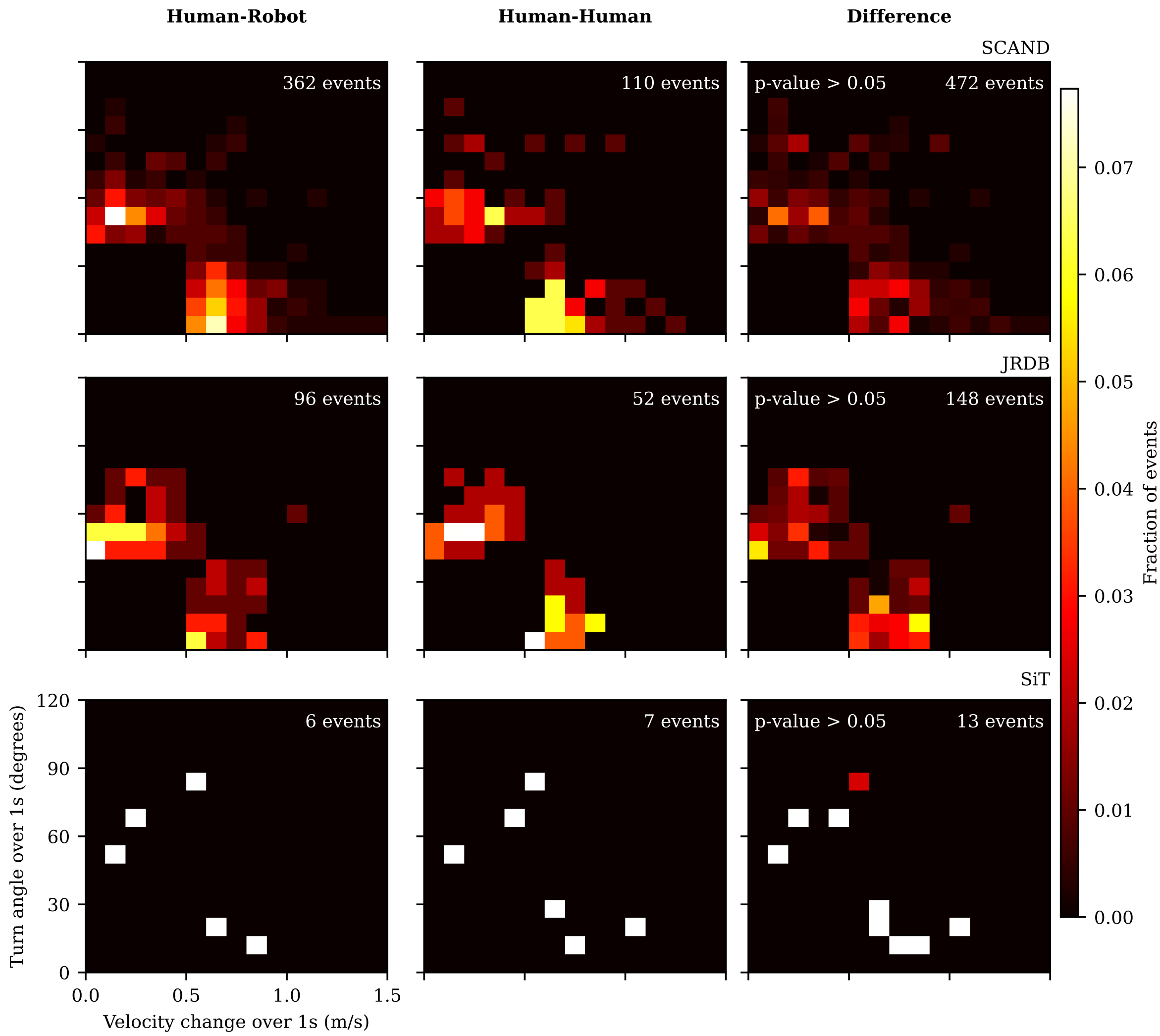

Figure S10: **Supplementary significant-event comparisons for SCAND, JRDB, and SiT.** Event maps are shown for HRI and HHI in the joint space of velocity change over 1 s and turn angle over 1 s, together with their residual maps. Across datasets, the event distributions remain qualitatively similar and do not indicate a clear HRI versus HHI difference.

Supplementary proxemics results for SiT

The SiT dataset yielded too few close-pass encounters for robust proxemics inference. We therefore report the available low-density results only for completeness (Figure S11), without drawing substantive conclusions from them. No usable robot-centered encounters were available for the higher-density strata, and even in low density the estimates are based on a small number of events. Consequently, the SiT proxemics results are too sparse to support reliable estimation of minimum-distance distributions, intrusion-count ratios, or density-dependent trends, and SiT is treated only as supplementary context rather than as evidential support for the main proxemics findings.

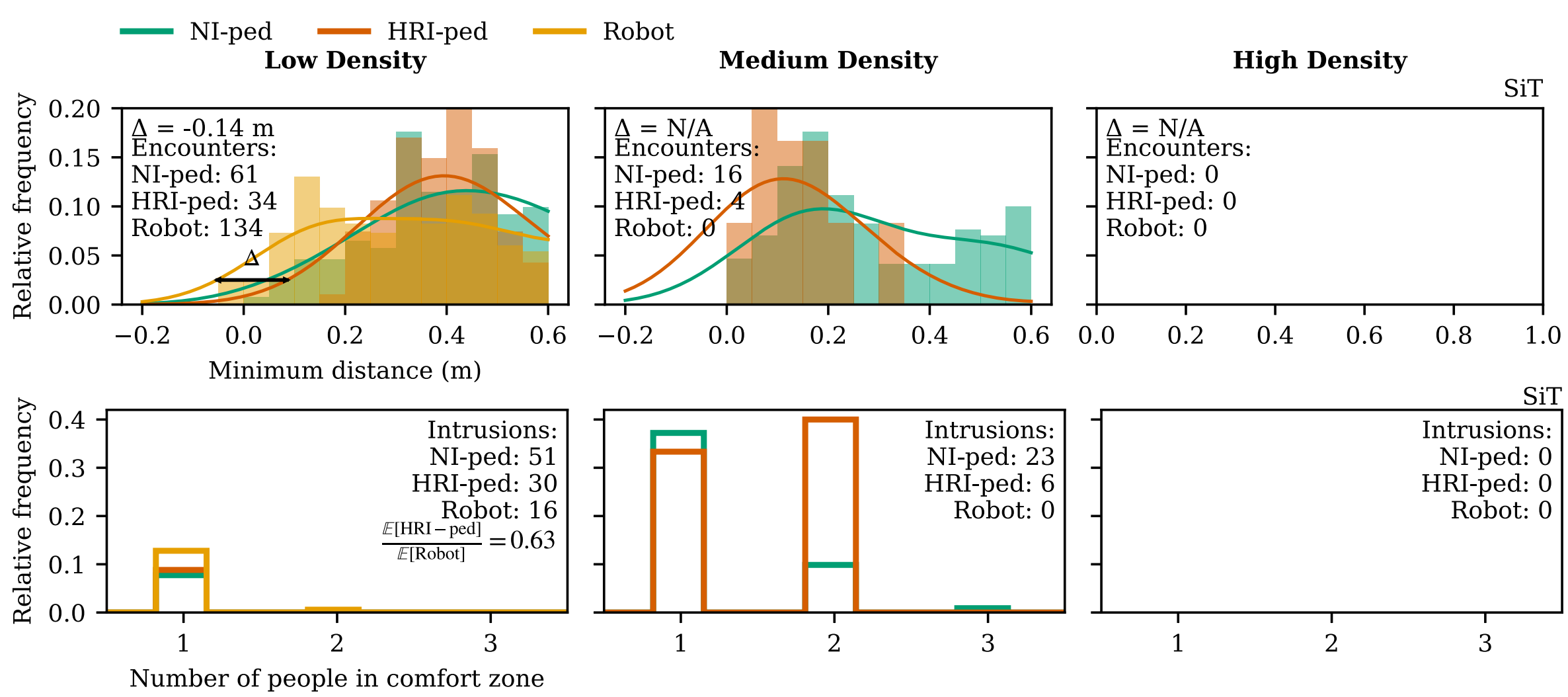

Figure S11: **Supplementary proxemics results for SiT.** Top row: minimum-distance distributions for NI-ped, HRI-ped, and Robot. Bottom row: intrusion-count distributions and the ratio of mean intrusion counts $\mathbb{E}[\text{HRI-ped}]/\mathbb{E}[\text{Robot}]$. These results are shown only for completeness. The small number of available encounters, together with the absence of usable higher-density robot-centered data, precludes robust estimation and does not support strong proxemics conclusions for SiT.

Other Supplementary Materials

Movie S1. Bird's-eye view visualization of robot navigation and interaction labeling in crowded public spaces. Top-down rendering of the Qolo robot in the CrowdBot-v2 dataset navigating through a crowd, providing an intuitive view of how interaction labels are generated over time. The video first introduces the visualization overlays (robot footprint and LiDAR points, near-collision zone, analysis radius used to filter distant pedestrians, and interaction-group labels for tracked pedestrians). It then plays a continuous segment of a recorded sequence, showing the robot's motion and how interaction labels evolve as pedestrians enter and leave the scene. Moving pedestrians and the robot are displayed with heading arrows and short trajectory traces.
